# Temporal Evolution of CO_2_ Conversion over Kaolin-Supported Ni, Ni–Ce and Fe–Cu Catalysts Under Dielectric Barrier Discharge Conditions

**DOI:** 10.3390/ma19132747

**Published:** 2026-06-26

**Authors:** Agata Dorosz, Michał Lewak, Katarzyna Jabłczyńska, Marta Mazurkiewicz-Pawlicka, Jakub Trzciński, Krzysztof Zaraska, Piotr Maćków, Jakub Jaworski, Arkadiusz Moskal

**Affiliations:** 1 Faculty of Chemical and Process Engineering, Warsaw University of Technology, ul. Waryńskiego 1, 00-645 Warsaw, Poland; michal.lewak@pw.edu.pl (M.L.); katarzyna.jablczynska@pw.edu.pl (K.J.); marta.pawlicka@pw.edu.pl (M.M.-P.);; 2 Centre for Advanced Materials and Technologies CEZAMAT, Warsaw University of Technology, Poleczki 19, 02-822 Warsaw, Poland; jakub.trzcinski@pw.edu.pl; 3 Łukasiewicz Research Network—Institute of Microelectronics and Photonics, Krakow Division, ul. Zabłocie 39, 30-701 Krakow, Poland; krzysztof.zaraska@imif.lukasiewicz.gov.pl (K.Z.); piotr.mackow@imif.lukasiewicz.gov.pl (P.M.)

**Keywords:** plasma-catalysis, CO_2_ conversion, metakaolin-based catalysts, surface metal redistribution, conversion dynamics, neural network modelling, predictive analytics, plasma-thermal synergistic effects, structure-activity relationship

## Abstract

**Highlights:**

- Kaolin acts as an active dielectric material, significantly influencing CO_2_ conversion in DBD reactors.
- Time-resolved analysis reveals transient behaviour and catalyst deactivation.
- Plasma–material interactions dominate over classical thermocatalytic effects.
- Time-series data enable application of machine learning for catalyst evaluation.

**Abstract:**

Carbon dioxide (CO_2_) conversion in non-thermal plasma is a promising route for carbon utilisation under mild conditions. This study investigates the performance and dynamic behaviour of kaolin-based catalysts modified with Ni (nickel), Ni–Ce (nickel-cerium), and Fe–Cu (iron-copper) oxides in a Dielectric Barrier Discharge (DBD) reactor. Materials were characterised using X-ray diffraction, energy-dispersive X-ray fluorescence, and scanning electron microscopy with energy-dispersive X-ray spectroscopy. CO_2_ conversion was evaluated at varying Plasma Energy Numbers (PEN = 1.65–20) with time-resolved gas analysis over a 10 min period. Results demonstrate that the kaolin support is not inert; its dielectric properties actively influence discharge characteristics. Ni-based catalysts exhibited the highest stable activity, reaching ~53% conversion for samples calcined at 500 °C. Conversely, adding cerium oxide significantly decreased conversion and induced temporal instabilities, contrasting with its typical role in thermal catalysis. Time-resolved measurements revealed that Ni–Ce and Fe–Cu systems exhibit initial activity followed by gradual deactivation, suggesting plasma-induced surface restructuring. These findings highlight that catalyst performance in DBD is governed by a complex interplay of chemical activity and plasma–material interactions. The generated time-series data provide a robust foundation for machine learning applications in predictive modelling and stability classification of plasma-catalytic systems.

## 1. Introduction

The development of a feasible “turnkey process” for carbon dioxide (CO_2_) conversion—one that seamlessly transforms atmospheric waste into value-added products—has become a cornerstone of modern sustainable engineering. The increasing concentration of CO_2_ in the atmosphere has driven extensive research into technologies enabling its efficient utilisation [1,2,3,4,5,6]. Conventional thermocatalytic processes for CO_2_ conversion typically require high temperatures and pressures due to the thermodynamic stability of the CO_2_ molecule, which limits their energy efficiency and practical applicability [3].

In this context, non-thermal plasma (NTP) technologies have emerged as a promising alternative, aiming to alleviate these energy barriers by enabling the activation of CO_2_ under relatively mild conditions [5,7,8,9]. Among various plasma configurations, the Dielectric Barrier Discharge (DBD) reactor is one of the most widely studied systems for gas-phase CO_2_ conversion. In DBD systems, energetic electrons can induce vibrational and electronic excitation, dissociation, and ionisation of CO_2_ molecules, providing a platform to unveil the complex interactions between plasma-generated species and catalytic surfaces.

However, despite these advantages, the overall energy efficiency and selectivity of plasma-based CO_2_ conversion remain key challenges. To address these limitations, plasma-catalysis has been proposed as a synergistic approach combining non-thermal plasma with catalytic materials [10,11,12]. In such systems, the catalyst may enhance the process by providing active sites for adsorption and reaction of plasma-generated species, as well as by modifying the local electric field and micro-discharge behaviour. Notably, in DBD reactors, the catalyst often plays a dual role: not only as a chemical catalyst but also as a dielectric material influencing the formation and distribution of micro-discharges.

This duality has sparked an ongoing debate over the mechanisms driving synergy, questioning whether the enhanced performance is driven by intrinsic surface chemistry or by the catalyst-induced restructuring of the plasma’s physical and electrical behaviour. In traditional thermocatalysis, nickel catalysts promoted by CeO_2_, as well as Fe- and Cu-based systems, are established benchmarks for CO_2_ activation [13,14,15,16]. While these formulations have been adapted for plasma-catalytic applications, research often focuses on their steady-state performance, leaving a gap in the understanding of their dynamic response [3,17,18,19,20,21]. Crucially, the introduction of such metallic active phases onto a dielectric support can fundamentally alter the discharge regime, making a transition to an efficient filamentary discharge more feasible—a decisive factor in determining the energy efficiency and stability of CO_2_ dissociation.

Beyond the active phase, the choice of catalyst support is a decisive factor for both performance and industrial viability. In this study, kaolin—a naturally abundant and inexpensive aluminosilicate—is utilised as a support. As clay minerals have garnered significant attention for their inherent economic and environmental attributes, kaolin offers a cost-effective alternative to expensive substrates such as carbon nanotubes or graphene. Its layered structure, thermal stability, and exceptional adaptability make it an ideal candidate for supporting nanocatalysts, facilitating the retrieval of active phases and promoting the adsorption of reactants within the plasma environment [22,23,24].

Crucially, in every variant of the investigated catalysts, the material is shaped into a specific hemispherical geometry. This intentional design represents a shift away from traditional “black-box” reactor studies towards a geometry-aware understanding of plasma–surface interactions. By utilising hemispheres, we aim to exploit the “edge effect” and non-uniform packing dynamics, which fundamentally alter the local electric field distribution and discharge behaviour compared to standard spherical or crushed packings.

However, a static understanding of these materials is insufficient, as plasma–catalyst systems are intrinsically non-equilibrium and time-dependent. While capturing these transients can be laborious, we employ time-resolved analysis of gas composition to unveil critical insights into the system’s evolution. This approach allows us to track catalyst stability and identify specific deactivation mechanisms, such as the potential accumulation of long-lived surface species. Furthermore, it enables the detection of potential “negative synergy” effects, where discharge intensification may inadvertently lead to surface poisoning. To quantify these energy-dynamic relationships, we introduce the Plasma Number (PEN), defined as the ratio of the input power to the product of the molar flow rate and the reaction enthalpy.

In this work, we investigate the performance and dynamic behaviour of kaolin-based catalysts modified with Ni, Ni–Ce, and Fe–Cu oxides in a DBD reactor. The complexity and non-linearity of these time-resolved data, particularly when correlating the Plasma Energy Number with conversion rates, suggest that traditional steady-state models are insufficient to fully describe the system’s evolution. The objectives of this study are threefold: (i) to evaluate the influence of catalyst composition on CO_2_ conversion, (ii) to elucidate the role of hemispherical kaolin pellets as both a catalyst support and a dielectric material, and (iii) to analyse the dynamic response of the system and evaluate the applicability of the Plasma Energy Number as a process descriptor. Furthermore, the collected time-series data are explored in the context of neural networks and machine learning applications, providing a basis for identifying hidden correlations and enabling future predictive modelling of these complex plasma-catalytic processes.

## 2. Materials and Methods

### 2.1. Materials

Nickel (II) nitrate hexahydrate (Ni(NO_3_)_2_ · 6H_2_O, pure grade), iron (III) nitrate nonahydrate (Fe(NO_3_)_3_ · 9H_2_O, pure grade, purity ≥ 98.0%), and copper (II) nitrate trihydrate (Cu(NO_3_)_2_ · 3H_2_O, pure grade, purity ≥ 99.0%) were obtained from Warchem Sp. z o.o. (Warsaw, Poland).

Cerium (III) nitrate hexahydrate (Ce(NO_3_)_3_ · 6H_2_O, purity 99.9%) was sourced from Pol-Aura (Olsztyn, Poland).

The kaolin (clay) powder (Nr CAS: 1332-58-7) was supplied by Biomus sp. z o.o. (Lublin, Poland).

Carbon dioxide of technical grade (99.5% purity, Product Code: 252158) was supplied by Air Products and Chemicals Inc. (Trexlertown, PA, USA).

Argon of Premier Grade 5.2 purity (99.9992%, Product Code: 168046) was supplied by Air Products and Chemicals Inc. (Trexlertown, PA, USA), with impurity specifications as follows: THC < 0.1 ppm, H_2_ < 2 ppm, O_2_ < 1.5 ppm, and N_2_ < 4 ppm.

A gas mixture of 2% hydrogen in argon (Inolinx^®^ TIG COM-X50S 200B, Product Code: 252079) was supplied by Air Products and Chemicals Inc. (Trexlertown, PA, USA). The mixture was prepared to high-purity standards suitable for precise plasma-catalytic applications, ensuring consistent composition and minimal impurity levels to maintain the integrity of the discharge environment.

### 2.2. Methods

#### 2.2.1. Preparation of Kaolin Support

The support material was prepared as hemispherical pellets with a diameter of 10 mm. Initially, a clay slurry was produced by dispersing kaolin powder in distilled water (binding agent). The mixture was transferred to custom-designed hemispherical moulds to ensure dimensional homogeneity and compactness. The resulting green bodies were air-dried for 24 h at 25 °C, followed by a secondary drying step at 80 °C for 12 h to enhance mechanical stability. Finally, the pellets were sintered in a muffle furnace at 950 °C. The temperature was increased at a constant rate of 4.5 °C/min and held at the target temperature for 2 h to produce the final ceramic support (Sample A0).

#### 2.2.2. Catalyst Synthesis

The active phases were deposited onto the kaolin hemispheres via wet impregnation method. For each batch, 130 g of kaolin pellets were immersed in 50 mL of an aqueous solution containing the appropriate metal nitrate precursors. The concentrations were calculated to achieve a final metal oxide loading of 5–10% wt. relative to the support mass, depending on the studied variant. The mixture was intensively stirred for 10 min to ensure uniform precursor distribution. Following impregnation, the samples were dried at 80 °C for 12 h.

The thermal treatment (calcination) was conducted in a furnace according to a specific multi-step temperature profile:

1. Heating from 25 °C to 200 °C at 2 °C/min, followed by a 1 h isotherm at 200 °C;
2. Heating from 200 °C to 400 °C at 1.5 °C/min, followed by a 3 h isotherm at 400 °C. Note: Sample B1’ was calcined at a higher temperature of 500 °C to investigate the effect of thermal treatment on metal-support interactions.

#### 2.2.3. Impregnation Strategies

Depending on the catalyst composition, three distinct synthesis strategies were employed:

- **Single-step Impregnation (B1, C1, C2):**
  Used for monometallic catalysts where a single precursor (Ni, Cu, or Fe) was dissolved in the solution.

- **Co-impregnation (B2, C3, C5):**
  The bimetallic (B2, C3) and trimetallic (C5) catalysts were prepared via simultaneous co-impregnation. The metal precursors were dissolved in a single aqueous solution to ensure the formation of highly integrated active phases and to maximise the interfacial contact between the metallic components.

- **Sequential Impregnation (B3, C4):**
  This method was employed to evaluate the effect of layered phase distribution. The support was first impregnated with the promoter (Ce or Fe), calcined according to the aforementioned profile, and subsequently impregnated with the active metal (Ni or Cu) followed by a final calcination.

The detailed composition and synthesis methods for all investigated samples are summarised in Table 1.

#### 2.2.4. Elemental Powder X-Ray Diffraction (XRD Analysis) and Rietveld Refinement

The crystalline structure and average crystallite sizes of the kaolin-based catalyst samples were determined by powder X-ray diffraction (XRD) using an XRDynamic 500 diffractometer (Anton Paar, Graz, Austria). The analysis was performed using Cu Kα radiation (λ = 1.5406 Å), with a monochromator utilised to ensure high spectral purity. Data acquisition was carried out over a 2θ range of 10–80°, employing Bragg–Brentano geometry and a continuous scan mode with a step size of 0.02°. For samples containing iron (Fe_2_O_3_), measurements were performed with the detector set to cut off radiation below 7.4 keV in order to suppress the strong fluorescence signal from iron. This configuration significantly improved the signal-to-noise ratio for iron-containing samples.

The quantitative phase analysis and crystallite size estimation were performed using the Rietveld refinement method implemented in the XRDanalysis software (version 1.2.3.3636, Anton Paar, Graz, Austria). The refinement procedure accounted for peak broadening related to both instrumental effects and the physical properties of the samples. For all samples, the kaolin phase present in the support was excluded from the refinement to focus on the active oxide phases (NiO, CeO_2_, CuO, or Fe_2_O_3_). The quality of the fit was monitored via the Bragg R-factor.

#### 2.2.5. Elemental Composition and Energy-Dispersive X-Ray Fluorescence Spectroscopy (EDXRF) Analysis

The elemental composition of the samples was evaluated by energy-dispersive X-ray fluorescence spectroscopy (EDXRF). The measurements were performed using an Epsilon 3^XLE^ spectrometer (Malvern PANalytical, Worcestershire, UK). The pelletised samples were ground in an agate mortar, and the resulting powder was transferred to a measurement cup covered with a Mylar (3.6 µm thick) film. The analysis followed the Omnian standardless procedure provided by the Epsilon 3^XLE^ software (version 1.4.G 8.34, Malvern PANalytical, Worcestershire, UK).

#### 2.2.6. Scanning Electron Microscopy with Energy Dispersive X-Ray Spectroscopy (SEM-EDS) Analysis

The morphology and atomic composition of the kaolin beads were investigated using a scanning electron microscope (SEM, SU8230, Hitachi, Tokyo, Japan). Samples of each material were mounted onto SEM stubs using conductive carbon tape. For topographical investigation, the beads were coated with a 10 nm layer of gold-palladium (80:20 ratio) using a sputter coater (Q150T, Quorum Technologies, Lewes, UK). Topography images were collected at an accelerating voltage of 10.0 kV and a working distance of 13.1 to 15.4 mm, utilising both upper and lower secondary electron detectors (SE(U/L)). Micrographs were recorded at a magnification of 5.00 k, to provide both representative overviews of the catalyst surface and detailed observations of morphological changes.

The surface atomic composition was determined via Energy Dispersive X-ray Spectroscopy (EDS) at an accelerating voltage of 30.0 kV and a working distance of 21.6 mm, using the upper detector on unsputtered (uncoated) samples to avoid interference from the gold-palladium signal.

#### 2.2.7. Characteristics of Non-Thermal Plasma Packed-Bed Reactor with Dielectric Barrier Discharge

**Figure 1:**
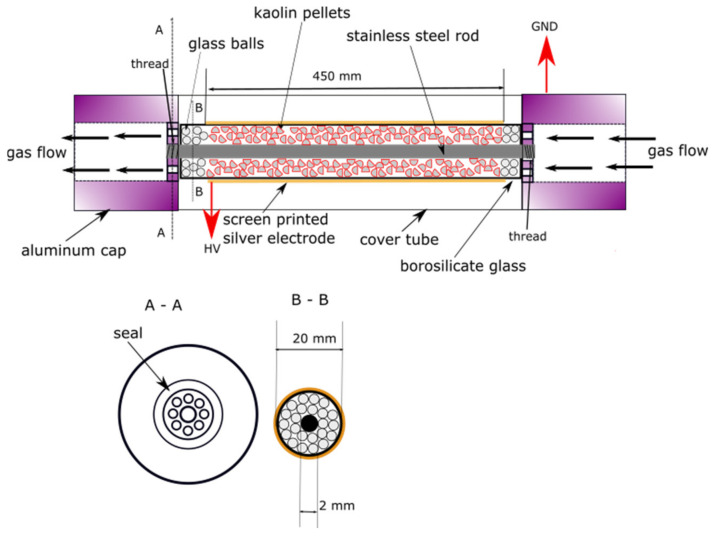
Schematic layout of the DBD-PB reactor featuring a screen-printed silver electrode: (A-A) transverse cross-section of the reactor’s holding assembly; (B-B) transverse cross-section of the primary reactor chamber.

Figure 1 provides a detailed schematic representation of the experimental reactor assembly. The reactor, operating in a dielectric barrier discharge packed-bed (DBD-PB) configuration, was constructed using a borosilicate glass tube (D_in_: 26 ± 1 mm, D_out_: 29 ± 1 mm, length: 500 ± 1 mm) as the working dielectric. In a departure from conventional designs, the outer high-voltage electrode was fabricated using a screen-printing technique, in which a conductive layer based on silver (Ag) nanoparticles was deposited directly onto the external surface of the glass tube. This process was carried out using a custom-built printer and a polymer-based printing paste, which was subsequently functionalised according to the manufacturer’s specifications and established thick-film technology protocols. Custom modifications to the electrode geometry and reactor configuration represent a key approach to adjusting the discharge characteristics and optimizing the overall process stability [25].

**Figure 2:**
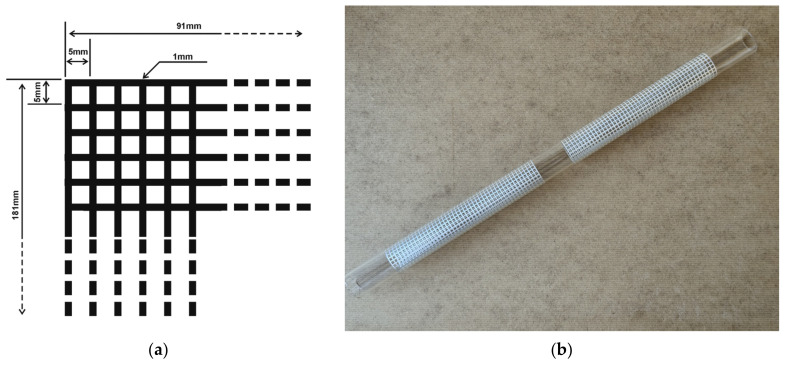
Conductor grid design for the plasma-catalytic reactor: (**a**) Schematic layout of the 4 mm × 4 mm mesh with a 1 mm conductive line width, indicating the radial direction along the tube and the azimuthal direction around the tube; (**b**) photograph of the glass tube with the printed conductive grid (tube view), showcasing the physical appearance of the patterned mesh.

The external electrode configuration consisted of two independent conducting grids, a design choice necessitated by the equipment’s maximum print size limitations. The grid pattern (Figure 2) featured 1 mm wide lines with a 5 mm step, resulting in 4 mm spacing in both directions. The grids were positioned 50 mm from each edge of the tube, leaving a 38 mm gap in the centre. This central gap was bridged with copper foil to ensure electrical continuity. A total of six identical tubes were prepared to ensure reproducibility. Compared to traditional metal foil electrodes, this screen-printing method ensured superior geometric precision and enhanced electrical contact—critical factors for maintaining discharge stability and homogeneity across the catalytic bed.

The grounded electrode was a 2 mm stainless steel rod axially aligned within the reactor. To ensure stable gas flow and prevent blockages, the terminal 25 mm sections were packed with inert glass beads (10 mm diameter); these zones remained electrode-free to isolate the ports from the discharge. The central active zone, defined by the annular space between the rod and the dielectric wall, was filled with the investigated material (A0, B1–B3, or C1–C5). For all trials, a constant mass of 130.0 g of pellets was used to ensure consistency.

#### 2.2.8. Experimental Setup and Plasma Generation Procedure

**Figure 3:**
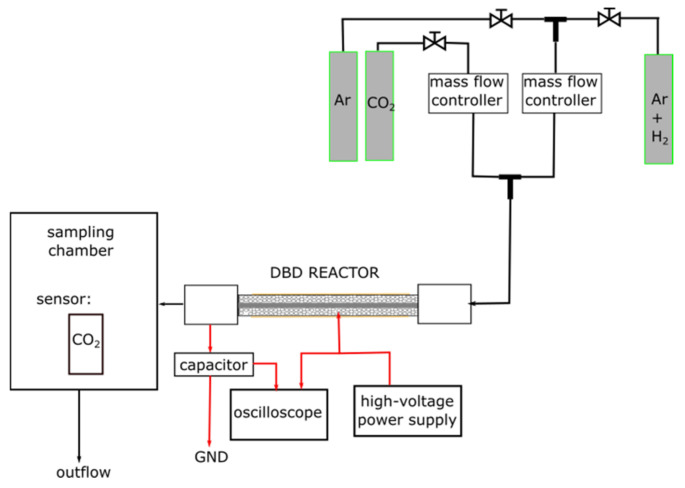
The schematic representation of the complete experimental setup. Red arrows marks the electric connections.

The experimental setup, illustrated schematically in Figure 3, was designed to facilitate precise control over gas mixing and plasma generation.

Various CO_2_ concentrations were obtained by mixing carbon dioxide with a constant argon flow of 5 dm^3^/min. The individual gas streams merged at a tee-junction to ensure uniform mixing before being fed into the DBD-PB reactor, which operated at atmospheric pressure.

Upon exiting the reactor, the effluent was directed into a sampling chamber for real-time monitoring of CO_2_ concentrations using a SparkFun SCD41 Qwii sensor (SEN-22396; Niwot, CO, USA). To ensure data reliability, these measurements were cross-validated via gas chromatography (Shimadzu GC-2014, Kyoto, Japan).

The operational procedure followed a rigorous cyclic routine. Following an initial in situ plasma-assisted reduction for 30 min using an H_2_/Ar mixture, the catalytic tests were performed. For each investigated CO_2_ concentration, once the gas flow reached a steady state, the plasma was ignited for exactly 10 min. Each measurement consisted of three consecutive plasma-on/plasma-off cycles to ensure reproducibility, with sufficient time allowed between intervals for the concentration to re-stabilise.

#### 2.2.9. Electrical Characterisation and Plasma-Flow Variants

The electrical parameters of the discharge were captured using a Rigol DHO804 high-resolution (12-bit) digital oscilloscope (bandwidth: 70 MHz, sampling rate: 1.25 GSa/s; Beijing, China) equipped with a Pintek HVP-39pro passive high-voltage probe (1000:1, 40 MHz; New Taipei City, Taiwan). Simultaneously, the grid power input was monitored in situ with a power meter. The plasma was sustained using a high-voltage AC power supply operating at a frequency of 24,000 Hz. The voltage amplitude and power input were adjusted according to the specific process requirements, resulting in three distinct plasma-flow variants:

- **Variant I:** CO_2_ flow rate of 0.013 dm^3^/min with a grid power input of 100 W and a peak-to-peak voltage of 6–7 kV.
- **Variant II:** CO_2_ flow rate of 0.039 dm^3^/min with a grid power input of 50 W and a peak-to-peak voltage of 6–7 kV.
- **Variant III:** CO_2_ flow rate of 0.078 dm^3^/min with a grid power input of 50 W and a peak-to-peak voltage of 6–7 kV.

During the preliminary reduction stage (using a 9 dm^3^/min H_2_/Ar mixture), the system operated at a higher grid power input of 130 W with a peak-to-peak voltage of approximately 9.5 kV to ensure effective activation of the packing material. Such systematic adjustments of discharge power, operating voltages, and gas flow rates represent a standard methodological approach to evaluating and optimizing the energy efficiency of plasma-driven CO_2_ decomposition systems [26].

#### 2.2.10. Data Reproducibility and Statistical Analysis

In total, ten distinct packing variants were evaluated, including the raw kaolin (A0) and the modified series (B1, B1’, B2, B3 and C1–C5). Each material was tested under the three plasma-flow variants described previously, resulting in 30 unique experimental conditions. To ensure robust results, each condition was investigated using a sequence of three consecutive plasma-on/plasma-off cycles. From these cycles, nine replicate measurements were extracted to determine the mean values and the associated standard deviations. This rigorous protocol was implemented to ensure the reproducibility of the plasma-catalytic performance across all investigated configurations.

#### 2.2.11. Energy Intensity and Plasma Energy Number

To evaluate the energy performance of the plasma-catalytic process across different experimental conditions, **a dimensionless plasma energy number (PEN)** was introduced. Standard metrics, such as Specific Energy Input (SEI), typically relate discharge power to the total volumetric flow rate. However, in systems with high dilution (such as the argon-rich environment used here), SEI may not fully reflect the energy intensity relative to the primary reactant. The plasma energy number addresses this by normalising the discharge power (P, [W])—calculated from the area of the Q-U Lissajous figures—against the molar flow rate of CO_2_ (*F*_CO_2_ inlet_, [mol/s]) and the theoretical enthalpy required for its decomposition:

PEN = *P*/(*F*_CO_2_ inlet_·Δ*H*^0^_298K_), (1)

where Δ*H*^0^_298K_ is the enthalpy for pure CO_2_ splitting, and is equal to 283 × 10^3^ J/moL.

The use of P instead of raw grid power accounts for the system’s electrical efficiency (equal to 50%). Here, the Lissajous figure is defined as a closed-loop function of the charge (Q, [C]) accumulated on the external capacitor versus the applied voltage (U, [V]), where the enclosed area represents the energy dissipated per single discharge cycle. The discharge power was subsequently determined as the product of this energy and the operating frequency. An exemplary Lissajous figure, illustrating the power determination methodology, is provided in Supplementary Material S.1, Figure S1.

Unlike the conventional Specific Energy Input (SEI), which relates power to the total volumetric flow rate, the PEN factor specifically assesses the energy surplus relative to the thermodynamic minimum required for the chemical transformation.

In this study, the calculated PEN values were approximately 20, 3.3, and 1.65 for Variants I, II, and III, respectively. This classification allows for a standardised comparison of the energy distribution across all investigated configurations, facilitating a rigorous assessment of how different packing materials (A0–C5) influence the discharge efficiency.

It should be noted that while the plasma was generated in a CO_2_/Ar mixture, the argon flow rate was kept constant across all experiments. Consequently, its influence on the electron energy distribution and charge-transfer processes is implicitly embedded within the fitted apparent parameters, and PEN serves strictly as an energy descriptor relative to CO_2_ throughput.

#### 2.2.12. Evaluation of CO_2_ Absolute Conversion

The performance of the CO_2_ dissociation process was evaluated based on the absolute conversion of the reactant. In this study, the absolute CO_2_ conversion (*X*_CO_2__, %) was calculated using the following equation:

*X*_CO_2__ = 100·(*C*_CO_2_ inlet_ − *C*_CO_2_ outlet_)/*C*_CO_2_ inlet_, (2)

where *C*_CO_2_ inlet_ is the inlet concentration of CO_2_ (ppm), and *C*_CO_2_ outlet_ is the outlet concentration of CO_2_ (ppm).

This definition provides a fundamental metric for assessing the total process efficiency, capturing the combined effects of the plasma discharge and the packing material within the reactor zone. To ensure comparability across all experimental variants—particularly those exhibiting transient behaviour or induction periods—the absolute conversion values and the subsequent thermodynamic efficiency calculations were consistently recorded exactly 10 min after plasma ignition. This protocol ensures that the data represents a stable period of reactor operation, allowing for a rigorous comparison between the different catalytic phases and energy regimes.

#### 2.2.13. Evaluation of Effective CO Splitting Energy Ratio (ESER)

Furthermore, the **Effective CO Splitting Energy Ratio (ESER)** was utilised as a complementary metric to evaluate energy efficiency. In this study, **ESER** is defined as the reciprocal of the plasma energy number:

ESER = 1/PEN,(3)

While PEN quantifies the energy intensity supplied to the reactor relative to the CO_2_ flow, ESER provides a direct measure of the process’s thermodynamic potential. This reciprocal relationship allows for a clear illustration of the trade-off between absolute conversion and energy utilisation; higher ESER values indicate a regime in which a larger proportion of the plasma energy is theoretically available for chemical transformation rather than being lost to thermal dissipation.

Conceptually, this approach facilitates an assessment of energy distribution at the molecular level. By multiplying ESER by the absolute conversion (*X*_CO_2__), it is possible to determine the specific fraction of energy successfully ‘invested’ into the chemical bonds of the reactant. Effectively, this represents energy coupled into each mole of CO_2_ that undergoes splitting, providing a clear distinction between productive chemical pathways and energy wasted on non-productive processes, such as gas heating or elastic collisions.

#### 2.2.14. Imaging of Discharge Propagation

The spatial distribution and discharge propagation within the reactor were documented using an iPhone 14 digital camera (Apple Inc., Cupertino, CA, USA). These visual observations were performed to examine the plasma–packing material interaction and to facilitate a qualitative assessment of the discharge modes.

## 3. Results and Discussion

### 3.1. Structural Characterisation via X-Ray Diffraction (XRD)

**Figure 4:**
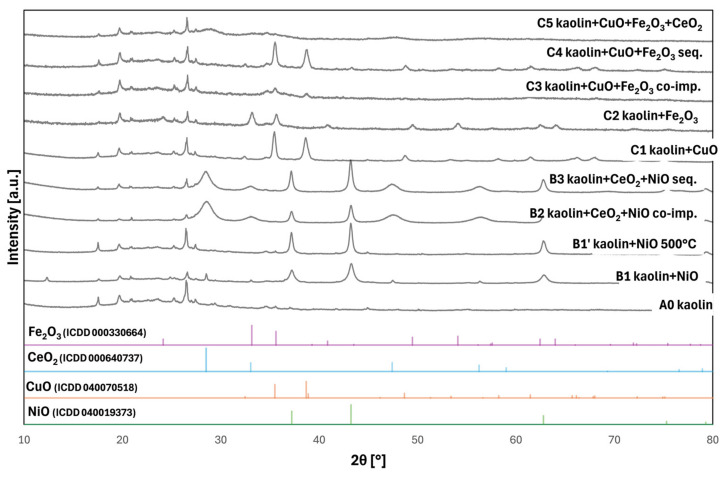
XRD patterns of the pristine kaolin support (A0) and metal-modified catalysts (B and C series), including reference patterns for NiO, CuO, CeO_2_, and Fe_2_O_3_ phase identification. Sample labels are positioned directly above their respective diffraction patterns. Sample labels are positioned directly above their respective diffraction patterns; “seq.” denotes sequential impregnation, while “co-imp.” refers to co-impregnation. The peaks were assigned to specific crystalline phases using ICDD reference standards: NiO (04-001-9373), CuO (04-007-0518), CeO_2_ (00-064-0737), and Fe_2_O_3_ (00-033-0664).

The phase composition of the synthesised materials was evaluated using powder X-ray diffraction (XRD). This analysis was conducted to characterise the crystalline framework of the kaolin-based support and to confirm the successful transformation of the nitrate precursors into their respective active metal oxide phases, specifically nickel oxide (NiO), copper oxide (CuO), cerium oxide (CeO_2_), and iron oxide (Fe_2_O_3_), following the thermal treatment. The XRD patterns of the raw kaolin support (Sample A0) and the synthesised catalyst formulations are presented in Figure 4.

The sample A0 serves as the reference material, representing the state of the kaolin support after thermal treatment at 950 °C prior to impregnation. The diffraction pattern of Sample A0 confirms the successful transformation of the raw kaolin into metakaolinite. According to the literature [27], heating kaolin to 950 °C results in complete dehydroxylation, in which the chemically bonded hydroxyl groups are removed, leading to the breakdown of the kaolinite crystalline structure. This is evidenced in Figure 4 by the absence of the characteristic kaolinite reflections at 12.3°, 20.1°, and 24.8° 2θ. Instead, Sample A0 exhibits a mineralogical composition dominated by an amorphous phase, attributed to metakaolinite, and residual crystalline impurities attributed to quartz and illite giving reflections at approximately 26.6° 2θ and around 17.6°, 19.8° 2θ respectively.

The XRD patterns for the catalyst formulations (Series B and C) reveal distinct crystalline phases corresponding to the deposited metal oxides, superimposed on the amorphous background of the metakaolinite support. The identification of these phases was performed by comparing the experimental data with references from the ICDD database, as shown at the bottom of Figure 4.

In the nickel-containing samples (B1, B1′, B2, B3), additional diffraction peaks were observed at approximately 37.2°, 43.2°, and 62.8° 2θ. These reflections can be attributed to NiO with a cubic (bunsenite) structure (ICDD card 04-001-9373). Notably, a weak reflection observed around 12° 2θ in the B1 sample can be assigned to residual kaolinite-like layered aluminosilicate domains, corresponding to the basal (001) reflection of kaolinite [25]. This peak is not associated with NiO, whose characteristic reflections occur at approximately 37.2°, 43.2°, and 62.8° 2θ. The presence of this weak low-angle reflection indicates that a minor fraction of the kaolin-derived support may retain local layered ordering after calcination. The additional weak reflections observed at approximately 28.6°, 33.1°, 47.6°, 56.4°, and 77° 2θ can be attributed to trace amounts of cubic CeO_2_ (fluorite structure). This minor cerium-containing impurity was most likely introduced during the preparation of the coating material. Due to the very low intensity of these peaks, the amount of CeO_2_ is considered negligible and does not influence the primary catalytic behaviour of the material. For the cerium-containing samples (B2, B3), further reflections appeared at 28.5°, 33.0°, 47.4°, and 56.3° 2θ, which are characteristic of CeO_2_ with a fluorite-type structure (ICDD card 00-064-0737). The relatively sharp peaks of both NiO and CeO_2_ indicate that these phases are well crystallised on the metakaolin support.

The catalysts in Series C show a more complex phase composition. In copper-containing samples (C1, C3, C4), diffraction peaks at 35.5° and 38.8° 2θ were assigned to CuO (tenorite, monoclinic structure; ICDD card 04-007-0518). Sample C5, although expected to contain CuO, did not show any distinct reflections, likely due to the high fluorescence background caused by the presence of iron.

For the iron-containing samples (C2, C3, C4, C5), Fe_2_O_3_ (hematite, ICDD card 00-033-0664) was considered as a reference phase. However, its characteristic reflections were difficult to clearly identify. This is likely due to strong X-ray fluorescence of iron under Cu Kα radiation, which increases the background and masks weaker diffraction peaks. The hematite phase was clearly identified only in sample C2, with reflections observed at 33.3°, 35.7°, 40.8°, 49.4°, 54.1°, 62.5°, and 64.0° 2θ.

The structural parameters and average crystallite sizes of the active phases, determined through Rietveld refinement, are summarized in Table 2.

The analysis provided detailed insights into the crystallinity and phase composition of the synthesized catalysts. For the B-series, the NiO phase consistently exhibited crystallite sizes ranging from 6.3 nm to 12.9 nm. Interestingly, in sample B1, the CeO_2_ phase showed significantly larger crystallites (51.7 nm) compared to samples B2 and B3, where the cerium oxide remained much more dispersed with crystallite sizes of 3.4 nm and 4.4 nm, respectively. This suggests that the specific preparation conditions for sample B1 promoted the grain growth of the cerium oxide phase.

In the C-series, the CuO phase in samples C1 and C4 demonstrated relatively large crystallite sizes of 67.9 nm and 81.0 nm, respectively, indicating a high degree of crystallinity for the copper oxide. In contrast, the Fe_2_O_3_ phase in sample C2 was characterized by a much smaller crystallite size of 10.7 nm. The refinement for samples C3 and C5 was not feasible due to insufficient peak intensity and significant peak broadening, which suggests that the active phases in these catalysts are either in a highly amorphous state or their crystallites are too small to be accurately measured by this method.

### 3.2. Elemental Composition via Energy-Dispersive X-Ray Fluorescence (EDXRF)

XRF analysis was performed post-reaction to determine the elemental composition of the catalysts after exposure to the plasma-catalytic process. As the Epsilon 3^XLE^ spectrometer is limited to the quantitative detection of elements heavier than sodium, the analysis focused on the transition metals and the aluminosilicate framework. Following the measurements, a post-processing calculation was performed to convert the raw elemental data into oxide weight percentages, assuming the formation of nickel(II) oxide (NiO), copper(II) oxide (CuO), iron(III) oxide (Fe_2_O_3_), and cerium(IV) oxide (CeO_2_).

The results of these calculations, compared against the nominal loadings, are summarised in Table 3. While the target loading for each oxide was 5 wt.% or 10 wt.%, the experimental values showed some deviations. Notably, the sequential impregnation method (sample B3) resulted in a significantly higher cerium content (17.09 wt.% CeO_2_) compared to the co-impregnated variant (B2), suggesting that the order of precursor deposition critically affects the final surface composition.

The aluminosilicate matrix, comprising aluminium (15.9–20.1 wt.%), silicon (21.2–24.3 wt.%), and potassium (1.7–2.3 wt.%), remained consistent across all samples, confirming the stability of the kaolin support during the modification processes. Detailed elemental data for the matrix are provided in the Supplementary Material S2.1, Table S1.

Furthermore, elemental analysis revealed that iron is an intrinsic component of the raw kaolin, with a baseline concentration of approximately 0.82 wt.% (expressed as 1.17 wt.% Fe_2_O_3_). It is presumed that these native iron species may possess inherent catalytic properties, potentially contributing to the overall performance of the support.

### 3.3. Surface Morphology and Elemental Composition via Scanning Electron Microscopy with Energy-Dispersive X-Ray Spectroscopy (SEM-EDS)

SEM-EDS analyses confirmed that the kaolin support retained its original character both after catalyst impregnation and following the plasma-catalytic decomposition of CO_2_, as presented in Table 4.

For all samples, oxygen, aluminium, and silicon remained the dominant constituents, with only moderate variations in their atomic fractions between the different formulations. This indicates that the applied modification routes primarily affected the surface distribution of the active metallic species rather than the bulk composition of the kaolin beads.

In the impregnated samples (Table 4), the O/Al/Si ratios remain close to those expected for kaolinite, with oxygen contents typically ranging between 48–58 at.% and the combined aluminium and silicon contribution accounting for approximately 25–35 at.%. Superimposed on this aluminosilicate matrix are the transition metals, which serve as the active phase. For instance, sample B2 shows clear incorporation of nickel and cerium, whereas in the C-series the relative contributions of Fe, Cu, and Ce vary with the specific impregnation strategy. The presence of potassium and calcium at levels of ca. 0.7–1.0 at.% is consistent with residual alkali and alkaline-earth impurities naturally occurring in kaolin.

A comparison of the pellets after plasma-assisted CO_2_ conversion (Table 3) with the starting materials reveals systematic changes in the surface distribution of the metallic elements. After the catalytic tests, the relative contribution of Si increases, while those of O and Al decrease. The pronounced drop in oxygen content may be attributed to the removal of surface hydroxyl groups from the kaolin framework under plasma conditions, a process that can alter surface chemistry and potentially affect the support’s catalytic activity. This indicates that the plasma process not only removes residual species but also modifies the accessibility and spatial distribution of the supported metal phase.

For the metal-containing samples, the trends depend on the specific formulation. In the B-series, post-plasma treatment of B3 leads to a markedly increased relative contribution of nickel (from 18.8 to 23.1 at.%), whereas in B2, a more stable distribution is observed. This behaviour, particularly evident in the Ni-Ce systems, is consistent with a partial redistribution or segregation of the active phase towards the outer surface during the plasma-catalytic process, enhancing the detectability of these metals within the interaction volume of the electron beam. In contrast, in the C-series, the plasma-driven process mainly affects the balance between copper and iron; for example, C1-Spent shows significant copper enrichment, while in C4-Spent, iron remains modest and copper dominates the metallic signal. These differences point to compositional and textural changes within the metallic phase, likely associated with redispersion or phase separation under plasma-thermal conditions. Moreover, the presence of carbon in the post-reaction samples could potentially indicate the accumulation of solid carbon products on the catalyst surface, a phenomenon typically observed in plasma-catalytic CO_2_ conversion. Notably, the A0 sample exhibits the lowest carbon content among all studied materials, suggesting that the kaolin support itself contributes minimally to carbon deposition. The addition of metal particles significantly influences the extent of carbon accumulation: the highest C deposits are observed for C3, in which Cu and Fe were co-deposited. The pronounced Fe signal detected on the surface of spent C3 pellets suggests that iron plays a key role in promoting carbon formation, possibly through catalytic pathways such as the Boudouard reaction or CO disproportionation. A comparison of single-metal samples (C1 and C2) further supports this interpretation—iron-containing catalysts consistently yield higher carbon deposits than their copper-containing counterparts.

EDS elemental mapping was added into Supplementary Material S3.1 (Figures S2–S15) to evaluate whether the plasma treatment caused uniform redistribution, local segregation, or agglomeration of the metallic phases. The maps, along with the corresponding statistical descriptors provided in Supplementary Material S3.2 (Table S2), show that the post-plasma changes are spatially heterogeneous, especially for the Fe–Cu-containing samples, supporting the conclusion that integral EDS values alone are insufficient for describing the surface evolution. Specifically, the quantitative data reveal that the Fe–Cu-bearing variants tend towards local segregation, characterised by larger, isolated element-rich clusters, whereas the Ni-based catalysts retain a higher density of smaller features across the surface.

### 3.4. Morphological Characterisation Utilising Scanning Electron Microscopy (SEM)

**Figure 5:**
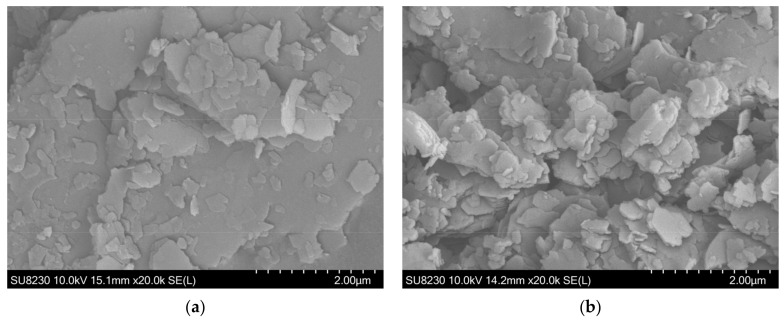
SEM micrographs of the pristine kaolin support (A0): (**a**) fresh state (calcined at 900 °C) and (**b**) spent state (following plasma-catalytic CO_2_ decomposition).

**Figure 6:**
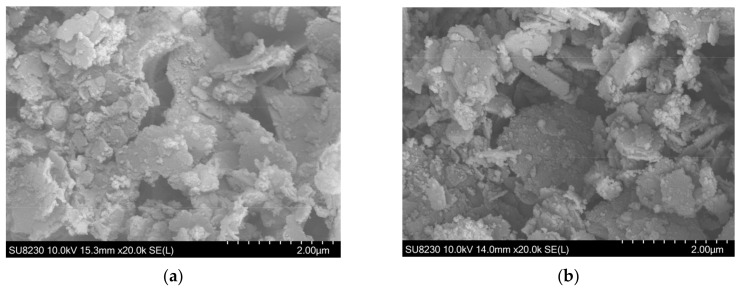

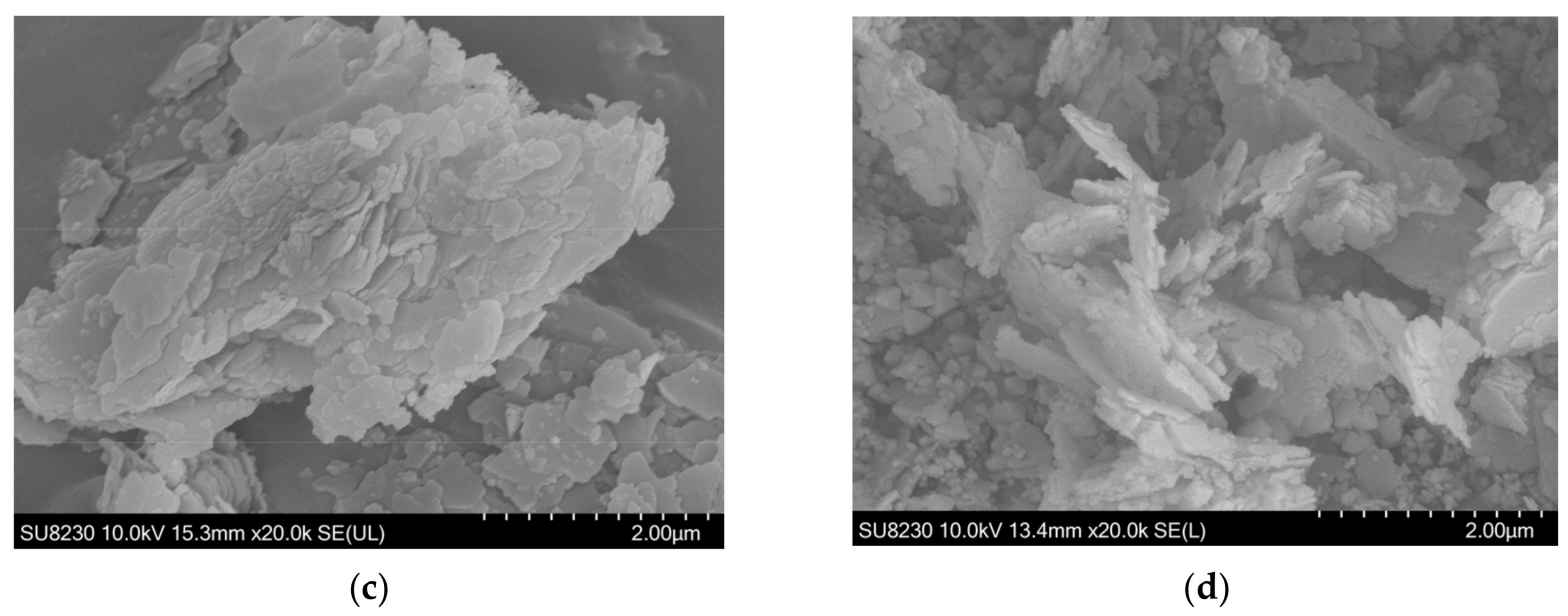
SEM micrographs of nickel-modified catalysts: (**a**) fresh B1 (calcined at 400 °C), (**b**) spent B1 (post-plasma catalytic CO_2_ decomposition), (**c**) fresh B1’ (calcined at 500 °C), and (**d**) spent B1’ (post-plasma catalytic CO_2_ decomposition).

**Figure 7:**
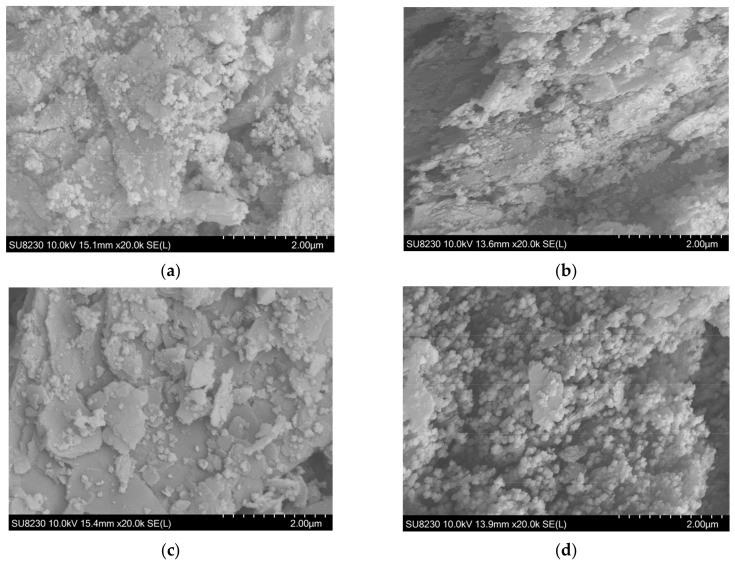
SEM micrographs of nickel-modified, Ce-promoted catalysts illustrating nodular agglomeration: (**a**) fresh B2 (co-impregnated), (**b**) spent B2 (post-plasma catalytic CO_2_ decomposition), (**c**) fresh B3 (sequentially impregnated), and (**d**) spent B3 (post-plasma catalytic CO_2_ decomposition).

**Figure 8:**
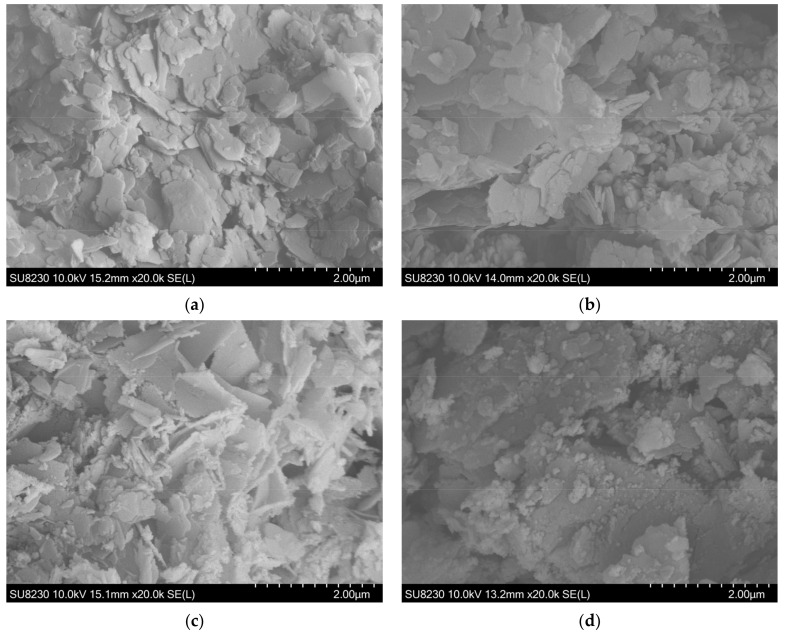
SEM micrographs of single-component copper and iron-modified catalysts: (**a**) fresh C1 (Kaolin + CuO), (**b**) spent C1 (post-plasma catalytic CO_2_ decomposition), (**c**) fresh C2 (Kaolin + Fe_2_O_3_), and (**d**) spent C2 (post-plasma catalytic CO_2_ decomposition).

**Figure 9:**
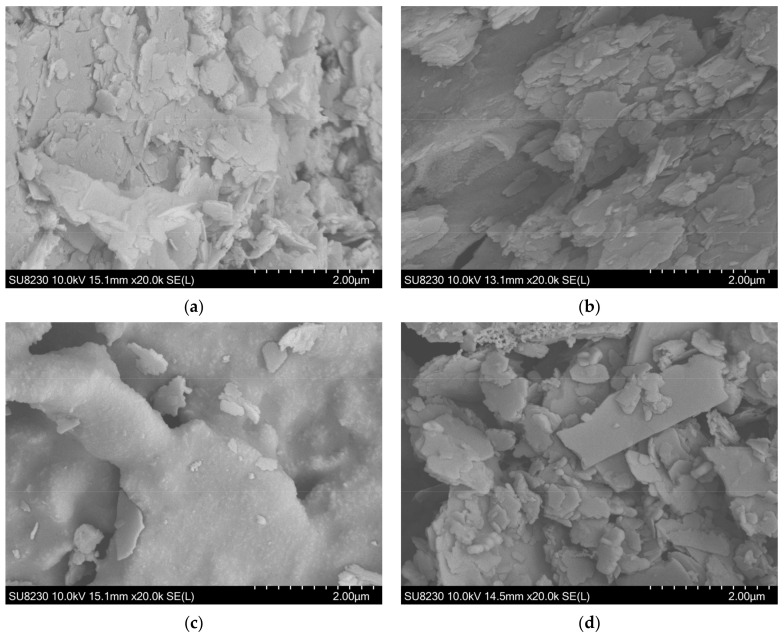
SEM micrographs of bimetallic Fe–Cu catalysts illustrating surface levelling and carbon deposition: (**a**) fresh C3 (co-impregnated), (**b**) spent C3 (post-plasma catalytic CO_2_ decomposition), (**c**) fresh C4 (sequentially impregnated), and (**d**) spent C4 (post-plasma catalytic CO_2_ decomposition).

**Figure 10:**
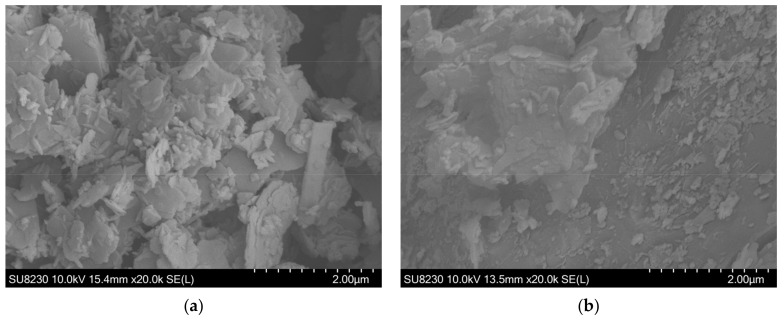
SEM micrographs of the multi-component Fe–Cu–Ce catalyst (C5): (**a**) fresh state and (**b**) spent state (post-plasma catalytic CO_2_ decomposition).

The surface morphology of the fresh and post-plasma catalysts was investigated by SEM to evaluate the influence of metal loading, impregnation strategy and plasma exposure on the kaolin-derived framework. Representative SEM micrographs are presented in Figure 5, Figure 6, Figure 7, Figure 8, Figure 9 and Figure 10. The detailed morphological assessment was carried out using images recorded at ×20.0 k magnification; the scale calibration of the images corresponded to a pixel size of approximately 2.480 nm px-1 and a field of view of approximately 6.35 × 4.76 µm. The dimensions of the structures should therefore be interpreted as apparent projected particle or agglomerate sizes visible in SEM micrographs, not as crystallite sizes.

The SEM analysis confirms that the investigated materials are not composed of isolated spherical nanoparticles. Instead, the catalyst surfaces consist mainly of strongly agglomerated lamellar, plate-like, flake-like, lath-like and, in selected samples, granular/nodular surface structures. A direct comparison of the fresh and post-plasma samples indicates that plasma exposure modifies the catalyst surface, although the extent of this modification depends strongly on composition and impregnation route. In most cases, the original support-derived lamellar framework remains visible, indicating that the plasma treatment does not cause complete structural collapse of the catalyst particles. Nevertheless, the post-plasma materials generally show increased surface roughness, more frequent fractured edges, local fragmentation of lamellar plates and a higher density of fine nodular or granular surface deposits.

Looking at specific compositional lines, the reference metakaolin support A0 proves highly stable; the fresh material features irregular plates and flakes (0.2–0.8 µm) forming larger aggregates (1.0–1.5 µm) alongside fine fragments (50–150 nm), and this primary framework is well retained in the spent sample, which exhibits only minor surface roughening and a slightly higher density of fine nodular/debris features in the 60–180 nm range (Figure 5a vs. Figure 5b).

In contrast, the nickel-containing catalysts display highly distinct, preparation-dependent structural responses. For the fresh B1 sample, the agglomerated flakes (0.5–1.5 µm) are decorated with fine nodules (40–120 nm). Upon plasma exposure, the basic framework is preserved, but the surface transitions to a more granular and heterogeneous state, where secondary particles grow to 70–200 nm and the finest surface nodules are reduced to 40–100 nm, reflecting moderate roughening and a distinct redistribution of the active phase (Figure 6a vs. Figure 6b). The most prominent restructuring among the nickel-only series is detected for the B1’ sample. Its fresh state is characterised by compact stacked lamellar aggregates (4.0–5.5 µm) with irregular plate-like flakes (0.5–1.5 µm). Following plasma treatment, the spent material displays a striking transition into an open, highly anisotropic radial and flower-like arrangement composed of platelet bundles (2.0–4.0 µm) and individual elongated laths (0.6–1.5 µm long, 80–250 nm wide), which points to significant fragmentation, exfoliation and structural rearrangement (Figure 6c vs. Figure 6d).

An opposite trend is observed for the B2 catalyst. The initially granular and nodular cauliflower-like aggregates of the fresh sample (0.5–1.5 µm) undergo partial compaction and surface restructuring during plasma exposure, resulting in a denser, ridge-like surface topography spanning extended rough regions of 2.0–5.0 µm (Figure 7a vs. Figure 7b). For the multi-metal B3 catalyst, severe surface roughening and particle fragmentation dominate; the fresh compact agglomerated flakes (0.7–2.0 µm) develop into a highly developed, cauliflower-like morphology where fine secondary nodules (50–180 nm) and finest features (30–80 nm) become dominant and cover the larger fragments (Figure 7c vs. Figure 7d).

Regarding the copper-promoted catalyst series, the structural modifications range from negligible to highly pronounced depending on the promoter composition. The single-metal C1 catalyst remains largely unaffected; its fresh agglomerated lamellar flakes (0.8–2.0 µm) show limited changes after the process, with the spent material exhibiting large flakes/agglomerates of 0.8–2.5 µm and minor fine debris of 80–200 nm (Figure 8a vs. Figure 8b). Conversely, a highly noticeable morphological transition occurs for the C2 sample. The fresh material is dominated by compact lamellar plates (1.0–2.5 µm) with broad smooth faces, but the post-plasma surface transitions to a mixed morphology where the dominant lamellar character is reduced, yielding large agglomerates (0.5–1.8 µm) rich in secondary fragments (100–300 nm) and fine granular clusters (40–120 nm) (Figure 8c vs. Figure 8d).

A more conservative behaviour is observed for the C3 and C4 catalysts, which successfully preserve their primary compact lamellar frameworks. For C3, the dense layered structure of the fresh plates (0.8–2.5 µm) develops additional edge roughness and elongated lath-like fragments (0.5–1.5 µm long, 100–300 nm wide) after plasma exposure (Figure 9a vs. Figure 9b). For C4, the large fresh aggregates (2.5–4.0 µm) remain clearly visible as large post-plasma platelets/agglomerates (1.5–3.5 µm, locally up to 5.0 µm) with only minor local nodular decoration and small attached flakes (Figure 9c vs. Figure 9d).

Finally, plasma exposure induces strong local variability in the C5 sample. The fresh layered aggregates (3.0–5.0 µm) composed of plates, flakes, and laths become increasingly heterogeneous, with the spent surface showing dense, fine granular/nodular regions (50–150 nm) coexisting right next to the larger remaining fragments (1.0–3.0 µm) (Figure 10a vs. Figure 10b).

These SEM results are highly consistent with the EDS observations discussed in Section 3.3, which reveal changes in the relative surface abundance of metallic elements and the appearance of carbon on the spent catalysts. Therefore, the plasma-induced surface restructuring, fragmentation and possible redeposition of fine secondary particles should be considered together with these surface compositional modifications. In particular, the increased roughness, granular/nodular deposits and local smoothing of some surface regions reflect a combined effect of active-phase redistribution, plasma-induced fragmentation and the deposition of carbonaceous species during CO_2_ plasma conversion.

Nevertheless, it should be emphasised that the present study focuses primarily on the temporal behaviour of carbon dioxide conversion in a dielectric barrier discharge (DBD) reactor filled with different kaolin-based catalytic packings. Therefore, comprehensive nanoscale and surface-chemical characterisation is beyond the scope of this work. Advanced analyses—including transmission electron microscopy (TEM) or high-resolution transmission electron microscopy (HRTEM) imaging, crystallographic plane and lattice spacing identification, alongside X-ray photoelectron spectroscopy (XPS) or high-resolution X-ray photoelectron spectroscopy (HR-XPS) of oxidation states, oxygen vacancies, and surface carbon species—are intentionally reserved for future studies dedicated to plasma-induced active phase transformations.

### 3.5. CO_2_ Absolute Conversion Under Different Plasma Conditions

**Figure 11:**
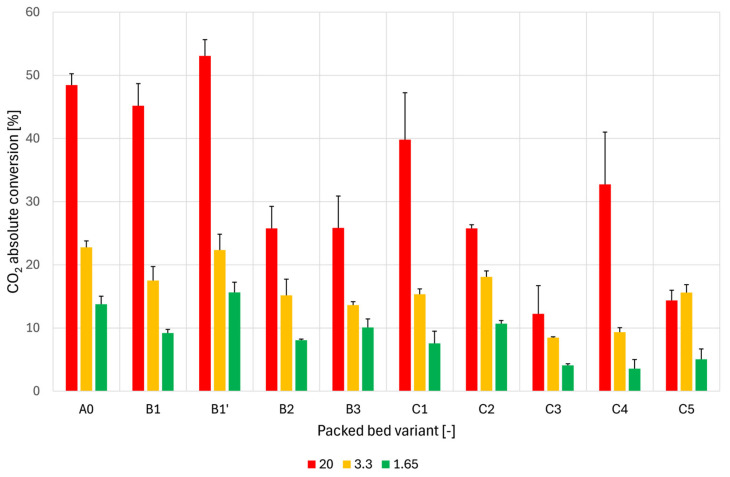
Steady-state absolute CO_2_ conversion (%) for pristine kaolin (A0) and metal-modified catalysts (B and C series) in the packed-bed DBD reactor at PEN = 1.65, 3.3, and 20.

The performance of kaolin-based materials and metal-modified catalysts was evaluated in a Dielectric Barrier Discharge (DBD) reactor under varying plasma energy numbers (hereafter referred to as PEN = 1.65, 3.3, and 20), as presented in Figure 11.

The results clearly indicate that CO_2_ conversion strongly depends on both the applied plasma energy and the nature of the catalytic material. At the highest investigated energy input (PEN = 20), significant differences in catalytic performance were observed, suggesting a transition to a regime in which plasma–material interactions dominate.

The highest CO_2_ conversion, reaching approximately 53%, was obtained for the Ni-modified kaolin sample calcined at 500 °C (B1′). Interestingly, the unmodified kaolin support (A0) demonstrated relatively high conversion, comparable to or exceeding several metal-containing systems. In contrast, catalysts containing combined metal oxides, particularly Ni–Ce (B2, B3) and Fe–Cu systems (C3, C5), showed significantly lower activity (~12–26%). At lower energy inputs (PEN = 1.65 and 3.3), the overall conversion decreased for all materials and the differences between catalysts became less pronounced, indicating that under low-energy conditions, the process is primarily governed by plasma chemistry rather than specific catalytic effects.

#### 3.5.1. Effect of Ni Incorporation and Calcination Temperature

The incorporation of Ni led to a noticeable enhancement of CO_2_ conversion, confirming its beneficial role in plasma-assisted processes. A significant effect of calcination temperature was observed (B1 vs B1′), with the higher temperature (500 °C) leading to a substantial increase in activity, as seen in Figure 11. This is attributed to changes in the physicochemical properties of the Ni phase. As confirmed by XRD analysis (Section 3.1), the nickel phase exists as well-crystallised NiO (bunsenite). The superior performance of B1’ suggests that the NiO species, possibly due to optimal grain size or interaction with the metakaolinite matrix, effectively alter the dielectric properties of the packed bed, enhancing micro-discharge formation and plasma–material coupling.

#### 3.5.2. Negative Impact of CeO_2_ Addition

Contrary to expectations from thermocatalytic systems, the introduction of CeO_2_ into Ni-based catalysts resulted in a significant decrease in CO_2_ conversion, as observed in Figure 11. This unexpected behaviour suggests that the role of CeO_2_ under plasma conditions differs fundamentally from its function in conventional catalysis. CeO_2_ may alter the dielectric properties of the bed, affecting the intensity of micro-discharges. Additionally, ceria is known to participate in redox processes that may facilitate the recombination of reactive oxygen species, effectively reducing the concentration of active intermediates available for CO_2_ conversion. Furthermore, SEM-EDS data (Table 4, Section 3.3) revealed a significant surface redistribution in sample B3 (Ni-Ce), where nickel content increased after the process; however, this was insufficient to counteract the suppressive effect of the ceria phase on the overall discharge chemistry.

The observed decrease in CO_2_ conversion highlights the intricate nature of plasma–catalyst interactions. While the aforementioned effects of the ceria phase on discharge behaviour and intermediate recombination are strongly indicated by literature trends, they remain tentative interpretations under the specific operating conditions of this study. Confirming the precise boundaries of these phenomena warrants further dedicated investigations, such as *operando* optical emission spectroscopy or dielectric permittivity measurements.

#### 3.5.3. Behaviour of Fe–Cu-Based Systems

Figure 11 shows that catalysts containing Fe_2_O_3_ and CuO exhibited highly variable performance. This instability is linked to the dynamic changes in surface composition under plasma conditions. The high fluorescence background in XRD (Section 3.1) and the pronounced surface enrichment of copper observed in SEM-EDS (e.g., C1-Spent, Table 4, Section 3.3) point towards a high mobility of the metallic phase. The presence of highly conductive metallic phases during operation leads to unstable catalytic performance, suggesting that Fe–Cu systems lack a stable active phase under these specific DBD conditions.

It should be noted that the reported values represent an apparent CO_2_ conversion calculated from the change in inlet and outlet concentrations. While CO, O_2_, and O_3_ are expected as the primary gaseous products under these conditions, the focus of this work is strictly on the dynamic, time-resolved response of the reactor rather than product selectivity or a complete carbon balance. A comprehensive quantitative assessment of product distribution and carbon balance remains outside the scope of this study and will be addressed in future work.

### 3.6. Time-Resolved Analysis and Dynamic Behaviour

**Figure 12:**
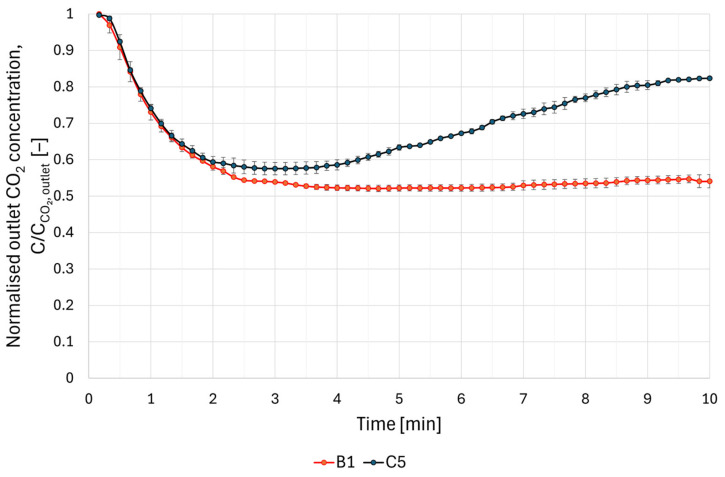
Evolution of the normalised CO_2_ concentration over time for B1 (NiO on kaolin, 400 °C) and C5 (CuO + Fe_2_O_3_ + CeO_2_ on kaolin) catalysts. The initial concentration (C/C_CO_2__ = 1) represents the CO_2_ outlet flow before plasma ignition.

As presented in Figure 12, time-resolved measurements revealed that while Ni-based catalysts reached a stable plateau (e.g., B1), the systems containing Fe and Cu exhibited pronounced transient behaviour (e.g., C5). An initial decrease in the CO_2_ concentration was followed by a gradual increase in the case of the C5 catalyst, indicating a loss of activity over time at a specific energy density (for PEN = 20). This phenomenon suggests that the system may be subject to complex transient effects, which could stem from either surface-related changes or fluctuations in the discharge stability. The SEM-EDS analysis of C5-Spent (Table 4, Section 3.3), which shows significant iron and copper redistribution, supports the hypothesis that structural reorganisation of the active phase occurs during the process, leading to deactivation. Such trends indicate that the interaction between the plasma and the multi-component C5 system (CuO + Fe_2_O_3_ + CeO_2_ on kaolin) is more dynamic than in the Ni-based catalysts. These observations demonstrate that steady-state metrics alone are insufficient for capturing the true behaviour of plasma-catalytic systems, particularly regarding stability-related phenomena.

### 3.7. Effective CO_2_ Splitting Energy Ratio Under Different Plasma Conditions

**Figure 13:**
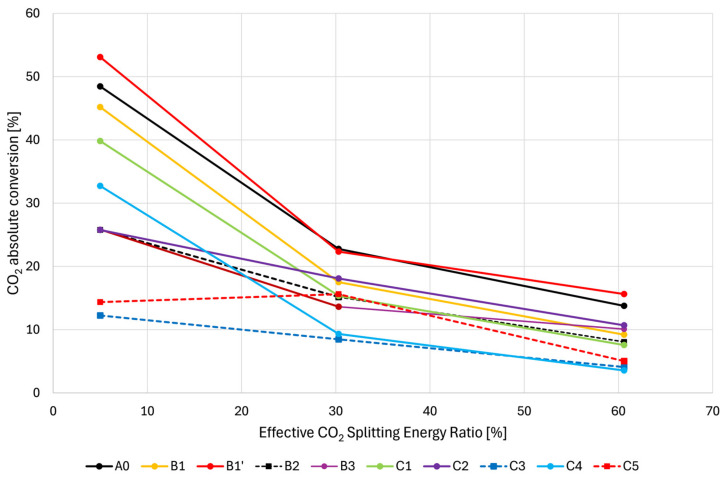
CO_2_ absolute conversion as a function of the Effective CO_2_ Splitting Energy Ratio (ESER) for various packed-bed reactor configurations.

The Effective CO_2_ Splitting Energy Ratio (ESER) is defined as the reciprocal of the PEN factor. The relationship between this ratio and CO_2_ absolute conversion reveals a characteristic trade-off. As shown in Figure 13, the highest conversion rates (reaching 53% for variant B1’) are achieved at the lowest ESER values (approximately 5%). This corresponds to the high-power regime, where increased energy density promotes higher dissociation while reducing energy utilization efficiency.

Conversely, the maximum ESER values—peaking at 60.5% for B1’ and 48% for A0—were recorded at the lowest power inputs (PEN = 1.65). In this regime, although the absolute conversion is lower, a significantly larger fraction of the plasma energy is effectively coupled into the chemical transformation of CO_2_ molecules rather than being dissipated as heat. The trade-off between energy efficiency and process yield is clearly illustrated by the ESER versus conversion profiles. While shifting to higher PEN values (lower ESER) leads to a substantial increase in absolute CO_2_ conversion, the diminishing returns observed in certain regimes suggest an upper limit for energy-intensive operation. For most variants, the most balanced performance—maximising conversion without a critical loss of efficiency—is observed in the intermediate ESER range, where the system avoids the excessive thermal losses typical of the high-power regime.

### 3.8. Discharge Characteristics and Energy Regimes

#### 3.8.1. Visual Observations and the Influence of Material Shaping

The physical interaction between the plasma and the packing material is governed by a complex interplay between the support’s geometry and its surface composition. Our visual observations confirmed that the specific properties of the kaolin support (A0) significantly shape the discharge characteristics.

**Figure 14:**
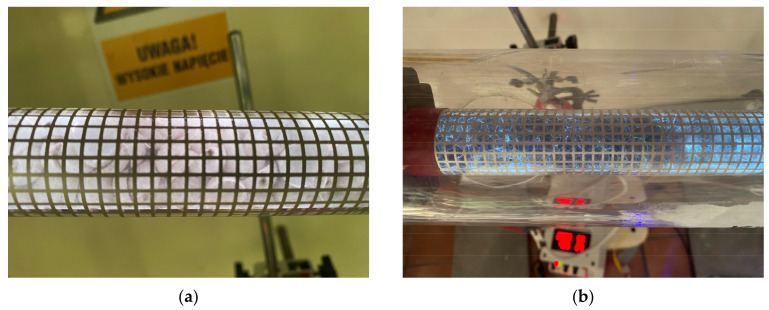
Visible plasma discharge within the dielectric barrier discharge (DBD) reactor filled with (**a**) pristine kaolin support (A0) at steady-state, (**b**) NiO/kaolin catalyst calcined at 400 °C (variant B1) at steady-state.

Regarding the discharge characteristics, the pristine kaolin A0 facilitated a stable, relatively homogeneous diffuse glow (Figure 14a). This behaviour is characteristic of a surface discharge over a dielectric material with a moderate dielectric constant. By distributing the electric field across the porous surface of the hemispheres, the kaolin ensures a uniform exposure of CO_2_ molecules to reactive species. This uniform plasma distribution is a key factor in the high energy efficiency observed for the A0 variant at lower PEN values (e.g., 1.65). Conversely, the introduction of Ni, Ce, and particularly Fe/Cu species triggered a notable regime transition to an intense filamentary discharge (Figure 14b). This mode is characterised by bright, high-current micro-discharges, indicating a concentration of plasma energy.

The transition to filamentary micro-discharges is primarily governed by local field enhancement, often dictated by metallic sites. As noted by Tu et al. [28], metallic clusters formed under plasma conditions act as primary discharge initiators. In our study, the conductive Ni, Ce, and Fe/Cu phases on the kaolin surface “short-circuit” the dielectric barrier effect, effectively forcing the plasma into aggressive discrete channels. According to Neyts et al. [29] and Michielsen et al. [30], the polarisation of dielectric pellets creates an enhanced electric field near the contact points. Our use of hemispherical geometry was a deliberate strategy to utilise this “edge effect”. Last, but not least, the random packing of these hemispheres was intended to optimise gas-surface interactions by increasing flow turbulence and tortuosity within the discharge gap, thereby extending the residence time of CO_2_ molecules in high-intensity zones.

#### 3.8.2. The Shift Between Plasma-Driven and Synergistic Regimes

The experimental data suggests a fundamental shift in the process-governing mechanism depending on the energy intensity, quantified by the PEN factor.

In the plasma-driven regime (PEN = 20), at high energy intensity, the system is dominated by a material-induced intensification of the discharge. For the C-series catalysts, the presence of pre-reduced, highly conductive metallic phases (Cu^0^, Fe^0^) dictates the spatial distribution of the electric field. These clusters act as focal points for localised field enhancement, forcing the discharge into an aggressive filamentary mode. This triggers micro-arcing and thermal stresses that exceed the material’s dissipation capacity, resulting in significant instabilities and large standard deviations.

Regarding thermal quenching and stability, this ‘plasma-driven’ instability is particularly pronounced under Variant I (high power, low CO_2_ throughput), where the lack of sufficient reactant flow prevents effective thermal quenching. Conversely, in the synergistic regime (PEN = 1.65 and 3.3), at lower energy inputs, the process shifts towards a balanced plasma-catalytic synergy. The higher concentration of CO_2_ acts as an effective quenching agent, dissipating excess energy and stabilising the discharge. This allows the chemical properties of the metal-support interface to play a traditional catalytic role while maintaining the structural and electrical integrity of the system.

### 3.9. Discharge Characteristics and Energy Regimes Mechanistic Aspects of Plasma-Catalytic CO_2_ Conversion

In the context of the investigated process, the dissociation of CO_2_ within the DBD reactor cannot be assumed to proceed via a single, straightforward elementary mechanism. Due to its high thermodynamic stability and linear structure, the activation of the CO_2_ molecule requires either high-energy inputs in the gas phase, specific interactions with surface active sites, or a simultaneous combination of both pathways. On solid catalyst surfaces, CO_2_ activation typically occurs through interactions with basic sites, surface defects, and oxygen vacancies, which serve as localized centres for adsorption and subsequent activation [31,32]. On oxide and aluminosilicate surfaces, CO_2_ undergoes chemisorption to form various surface intermediates, such as carbonates, bicarbonates, or activated linear/bent CO_2_^δ−^ species. Carbonate forms generally arise from the interaction of CO_2_ with basic surface oxygen atoms of the oxide, whereas bicarbonate species are frequently linked to the presence of surface hydroxyl groups. When surface defects or oxygen vacancies are accessible, a partial charge transfer to the CO_2_ molecule can take place, inducing a bent geometry that weakens the C=O bonds and significantly enhances its reactivity [31,33].

For the kaolin support—an aluminosilicate matrix—surface hydroxyl groups and active sites associated with framework silicon and aluminium atoms play a key role. Literature regarding gas adsorption on kaolinite indicates that this material exhibits a higher adsorption affinity for CO_2_ than for CH_4_ and N_2_. Du et al. [34] demonstrated that the adsorption affinity follows the sequence N_2_ < CH_4_ < CO_2_, suggesting that the kaolin surface inherently promotes a localized enrichment of CO_2_ molecules in the vicinity of the active zones. The incorporation of transition metal oxides, such as NiO or CuO, further modifies the surface properties of the packing by altering the density and chemical nature of these active sites. These metal oxides introduce additional basic sites, redox centres, and surface defects that directly govern the pathways of CO_2_ chemisorption. For instance, in CuO-containing systems, the presence of the copper oxide phase has been shown to strongly influence the activation and conversion trends of CO_2_, impacting both product selectivity and the precise nature of the reactive surface states [33]. Broadly, transition metal oxides are widely recognized as vital components for enhancing CO_2_ capture, adsorption, and subsequent conversion [32].

However, within a packed-bed DBD reactor, the conversion mechanism is fundamentally more intricate than in conventional thermocatalysis. The non-thermal plasma provides high-energy electrons capable of inducing vibrational and electronic excitation, ionization, and direct electron-impact dissociation of CO_2_ in the gas phase. Concurrently, the surface of the packing framework adsorbs CO_2_ and stabilizes these reactive intermediates. Therefore, the observed temporal evolution of CO_2_ conversion is likely the result of an overlapping dual phenomenon: gas-phase plasma activation combined with surface-level chemisorption on the kaolin support and the loaded metal oxides. Accordingly, the proposed kinetic model must be understood as an apparent model describing the global macroscopic response of the plasma-catalytic system, rather than a definitive elementary reaction mechanism.

The mechanistic interpretation proposed in this work should be regarded as a literature-supported hypothesis explaining the observed temporal behaviour of CO_2_ conversion under DBD conditions. A complete elucidation of the elementary reaction pathways requires dedicated plasma diagnostics and surface-sensitive operational studies, which are beyond the scope of the present work.

### 3.10. Mathematical Modelling

#### 3.10.1. State of the Art

The conversion of carbon dioxide in low-temperature plasma generated by dielectric barrier discharge (DBD) has emerged as one of the most intensively studied pathways for plasma-assisted CO_2_ valorisation. This interest is driven by the possibility of activating the highly stable CO_2_ molecule under near-atmospheric conditions and at moderate gas temperature, while using electrical energy as the driving force for chemical transformation. In DBD systems, energy is transferred primarily to electrons rather than to bulk gas heating, which creates favourable conditions for non-equilibrium activation of CO_2_. Owing to their simple design, atmospheric-pressure operation, and potential scalability, DBD reactors are increasingly considered promising platforms for future power-to-chemicals and power-to-fuels technologies [35]. Despite extensive experimental investigation, the mechanisms governing CO_2_ dissociation in DBD reactors remain insufficiently understood. This is mainly due to the multiscale nature of the process, in which plasma chemistry, electrical phenomena, energy transfer, gas flow, and reactor geometry are strongly coupled. Therefore, mathematical modelling has become an essential tool for interpreting experimental observations and identifying the dominant factors controlling conversion and energy efficiency.

Over the last decade, modelling approaches have evolved from simplified global descriptions to increasingly sophisticated fluid- and reactor-scale models that account for the spatiotemporal non-uniformity of discharge, transport processes, and plasma–material interactions [36,37]. One of the major trends in this field has been the transition from zero-dimensional and global-kinetic approaches to time- and space-resolved fluid models. A representative example is the work of Ponduri et al. [36], who developed a one-dimensional, time-dependent fluid model for CO_2_ dissociation in a symmetric DBD reactor, including an extensive vibrational kinetics scheme. Their study demonstrated that the positive and negative half-cycles of the applied voltage are not equivalent and result in spatially asymmetric distributions of stable products, such as CO and O atoms. More importantly, they showed that CO formation occurs predominantly during the discharge pulses and is mainly governed by electron-impact dissociation. They also found that the calculated CO_2_ conversion increases almost linearly with specific energy input, consistent with experimental trends. The growing importance of integrated macroscopic descriptors constitutes another clear trend in the modelling of DBD-based CO_2_ conversion. Among these descriptors, the specific energy input (SEI) is among the most widely used parameters for correlating reactor performance with operating conditions. In the study by Ponduri et al., SEI was identified as a robust scaling parameter for CO_2_ conversion, largely independent of the specific means by which it is achieved, including variations in voltage, frequency, residence time, or dielectric thickness. This observation is particularly important from a modelling perspective because it provides a physical basis for developing reduced-kinetic or semi-empirical models in which the overall transformation rate is correlated with energy-related process parameters rather than with a detailed description of each microdischarge event [36].

A parallel line of research has focused on identifying the dominant reaction pathways and reducing the complexity of plasma-chemical schemes. In their combined experimental and computational study, Aerts et al. [35] showed that although a detailed kinetic description of CO_2_ splitting in DBD can involve dozens of species and hundreds of reactions, reduced reaction sets can still capture the essential behaviour in restricted conversion ranges. Their analysis indicated that in DBD reactors, the key processes responsible for CO_2_ splitting are direct electron-impact dissociation, electron-impact ionisation followed by dissociative recombination, and dissociative electron attachment. At the same time, they demonstrated that backward reactions, especially the recombination of CO with oxygen-containing species, become increasingly important at longer residence times and higher SEI, thereby contributing to the experimentally observed saturation of conversion. Their work also highlighted that a simplified model may remain valid only at relatively low conversion, whereas higher-conversion conditions require more complete reaction schemes [35].

Another important development has been the extension of plasma-chemical models toward reactor-scale flow descriptions. Kotov and Koelman [37] proposed a one-dimensional plug-flow reactor model for plasma-chemical conversion of CO_2_, coupling reaction kinetics with species transport and energy balances along the reactor axis. Their analysis revealed that even when a substantial fraction of the input power is initially deposited into vibrational excitation, rapid vibrational–translational (VT) relaxation can strongly redirect this energy toward heating of the heavy particles. As a result, CO_2_ dissociation in the model proceeds predominantly through thermal quenching pathways rather than through an idealised vibrational ladder-climbing mechanism. They further showed that mitigating VT losses requires a substantial increase in specific input power, and that even under such conditions, the overall energy efficiency becomes constrained by reverse reactions converting CO back to CO_2_. These findings significantly shifted the focus of reactor modelling from vibrational excitation alone toward a broader analysis of energy redistribution and competing backward processes [37].

In recent years, the role of reactor geometry and dielectric materials has also become increasingly prominent in mathematical descriptions of DBD systems. This is especially relevant in packed-bed reactors, where the inserted solid phase cannot be regarded as an inert structural element. Van Laer and Bogaerts [38] demonstrated, using a fluid model of a packed-bed DBD reactor, that the dielectric packing strongly modifies the local electric field through polarisation effects. Their simulations showed that contact points between beads and between beads and walls become regions of enhanced electric field and elevated electron temperature and therefore act as preferred ignition sites for plasma formation. They further concluded that at low applied voltage, the discharge remains confined to these contact regions and exhibits Townsend-like characteristics, whereas at higher voltage, it can propagate through void channels between the beads, approaching a glow-like behaviour. A particularly important methodological conclusion of this work is that realistic packed-bed modelling must include not only the dielectric contacts but also the so-called channel of voids, as this feature is essential for enabling plasma propagation across the reactor gap [38].

The most recent trend in the field is the development of multidimensional models that couple plasma transport with fluid flow and reactor design. Mas-Peiro et al. presented a two-dimensional model of a DBD reactor using argon as the diluent gas, integrating plasma physics with laminar-flow calculations in COMSOL® Multiphysics Version 6.1. Their study emphasised that moving from 0D or 1D descriptions to 2D geometry with fluid flow imposes a necessary compromise in chemical detail, yet it provides valuable insight into the effect of design variables such as inlet flow rate, dielectric thickness, dielectric material, and reactor length. The authors showed that such models can reproduce key plasma characteristics, including electron density, ion and excited-species distributions, and mean electron energy, while simultaneously linking them to flow behaviour inside the reactor. They also pointed out that the inclusion of fluid dynamics opens a pathway to reactor-level optimisation, even though simplified chemical and geometric assumptions remain necessary at this stage [39].

Taken together, the available literature shows a clear evolution in the mathematical modelling of CO_2_ dissociation in DBD reactors. The field has progressed from global descriptions toward fluid and reactor-scale approaches; from purely kinetic formulations toward coupled descriptions of chemistry, transport, and electric field effects; and from idealised empty-gap reactors toward more realistic geometries involving dielectric packing and gas flow. At the same time, the literature consistently indicates that CO_2_ conversion in DBD cannot be understood solely in terms of reaction chemistry. Instead, the process is governed by a complex interplay between electron-driven kinetics, vibrational and thermal energy transfer, reverse reactions, reactor hydrodynamics, and the local electric-field distribution shaped by geometry and dielectric properties [35,36,37,38,39].

#### 3.10.2. Development of the Reaction Kinetic Model—Non-Linear Regression

In this context, there is a strong motivation to develop reduced yet physically interpretable kinetic descriptions that preserve the dominant process dependencies while remaining suitable for reactor-scale analysis and data-driven tools. Such an approach is particularly useful when the objective is not only mechanistic interpretation but also parameter estimation, comparison of catalyst or packing effects, and future integration with machine learning methods for predicting and classifying reactor behaviour. To determine a kinetic model that mathematically describes changes in carbon dioxide concentration at the reactor outlet, a black-box model was employed. This approach was dictated by the lack of a clearly defined mechanism for carbon dioxide decomposition in low-temperature plasma. Our previous attempts to determine a simple kinetic model for this reaction [22] indicate that, for a packed bed composed of materials with the same chemical composition and a similar particle shape, an nth-order kinetic model may be applied, provided that low-temperature plasma is generated throughout the entire reactor volume. To verify the experimental results, an algorithmic procedure was used to select an appropriate kinetic model for the reaction. During data preparation, particular emphasis was placed on the goodness of fit of the data to the following relationship:

d*y*_CO_2__/d*t* = *k*_0_·exp(*E*_act_/PEN) · y_CO_2__*^n^*, (4)

where

- d*y*_CO_2__/d*t* is the reaction rate [s^−1^];
- *y*_CO_2__ is the molar fraction of carbon dioxide;
- *k*_0_ is the pre-exponential factor (frequency factor) [s^−1^];
- *E*_act_ is the dimensionless apparent activation energy of the process;
- *n* is the dimensionless empirical fitting parameter.

As shown by Dorosz et al. [22], such a simple model can be used to preliminarily estimate changes in reactant concentration at the outlet of a DBD reactor. In Equation (4), the effect of the plasma energy number, PEN, differs from the expected effect of temperature in chemical reaction processes. This approach was adopted because it provided a better fit of the model to the experimental data. Placing the PEN factor in the numerator (see relationship (4)) yielded negative activation energy values from the non-linear regression.

It should be emphasised that the parameter *E*_act_ obtained from fitting the proposed kinetic equation is purely empirical in nature. Consequently, it should not be interpreted as an activation energy in the physicochemical sense commonly associated with classical kinetic models. Negative values of *E*_act_ should therefore be understood only as a result of the regression procedure leading to the best fit of the model to the experimental data. Similarly, the adopted form of the equation, in which PEN appears in the exponential term, does not follow from a mechanistic derivation but was selected exclusively on the basis of approximation quality. For this reason, the proposed equation should be regarded as an empirical correlation useful for quantitative description of the experimental trends rather than as a direct representation of the underlying elementary reaction mechanism.

Using data on the variation in carbon dioxide concentration at the reactor outlet over time, the derivative describing the CO_2_ decomposition rate was calculated numerically from the following differential equation (see Equation (5)). The mole fraction values at the nodal points were calculated using spline interpolation. The entire procedure was implemented in MATLAB R2025b. The derivative was approximated using a fourth-order finite-difference scheme, O(h^4^), with a differentiation step of ∆t_i_ = 10^−9^ s:

(5) $$\frac{d}{dt}\left(y_{{CO}_{2}}(t_{i})\right)\approx \frac{8\cdot y_{{CO}_{2}}\left(t_{i}+∆t_{i}\right)-8\cdot y_{{CO}_{2}}\left(t_{i}-∆t_{i}\right)-y_{{CO}_{2}}\left(t_{i}+2\cdot ∆t_{i}\right)+y_{{CO}_{2}}\left(t_{i}-2\cdot ∆t_{i}\right)}{12\cdot ∆t_{i}},$$

The experimental signals were recorded with a sampling interval of 10 s. The experimental dependence of the CO_2_ mole fraction on time was approximated using a cubic spline, and the reaction rate was evaluated as the derivative of this spline-approximated function. In the numerical implementation, a small internal increment of 10^−9^ s was used only for the finite-difference evaluation of the derivative of the smooth spline function. This value was not associated with the experimental acquisition frequency and should not be interpreted as the measurement time step. Since the calculations were performed in MATLAB using double-precision arithmetic, which provides approximately 15–16 significant decimal digits, this internal numerical increment can be represented computationally. However, the physical time increment of the experimental data remained equal to 10 s.

**Figure 15:**
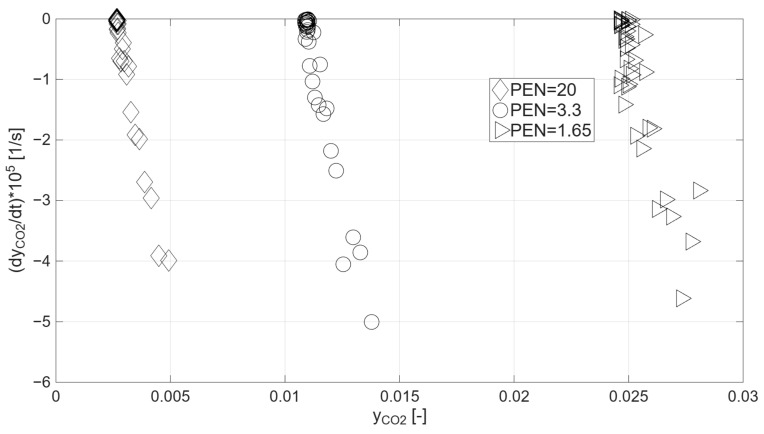
Effect of carbon dioxide content on the CO_2_ decomposition rate—Variant A0 (Pure Kaolin).

Figure 15 shows changes in the CO_2_ decomposition rate as a function of the gas mole fraction. This comparison was made for three different values of the power number, PEN, which characterises the plasma quality. As shown, the power number directly affects the parameters that describe the kinetic model. The presented measurements were carried out in a reactor packed with Pure Kaolin—Variant A0.

**Figure 16:**
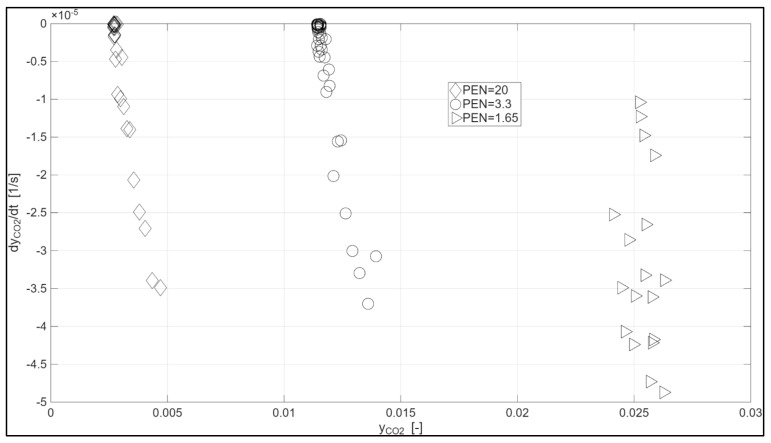
Effect of carbon dioxide content on the CO_2_ decomposition rate—Variant B1 (Kaolin + 10 wt.% NiO).

The next plot (see Figure 16) compares changes in the CO_2_ decomposition rate as a function of CO_2_ mole fraction for a kaolin packing material consisting of 10 wt.% nickel oxide. The plot indicates that, in the initial stage, the reaction rate is slightly lower for this packing than for the packing shown in Figure 15. Interestingly, at the lowest power number, PEN = 1.65, the initial reaction rate is slightly higher than that observed at higher PEN values. This difference may be attributed to the different type of packing material used. This suggests that the presence of nickel oxide may influence the plasma–surface interactions and, consequently, the apparent kinetics of CO_2_ decomposition. Therefore, the packing composition should be considered an important factor affecting both the reaction rate and the overall performance of the DBD reactor.

**Figure 17:**
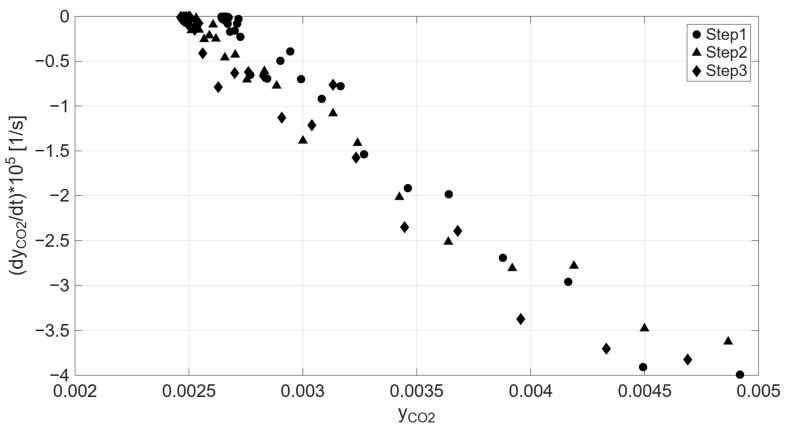
Effect of carbon dioxide content on the CO_2_ decomposition rate—Variant A0 (Pure Kaolin, PEN = 20).

To verify the repeatability of the experimental results, each plasma generation process was carried out three times under identical conditions. Figure 17 presents the experimental results showing the effect of the carbon dioxide mole fraction on the rate of its decomposition. All three measurements (Step 1, Step 2, and Step 3) were performed at the same PEN and with an identical molar flow rate of CO_2_. The temperature and pressure conditions did not change significantly between successive measurements.

Figure 17 shows that the kinetic reaction model (see Equation (4)) can be applied to describe the course of CO_2_ decomposition in the DBD reactor. Secondly, the repeatability of the measurement results is not ideal, most likely due to non-uniform plasma generation in the reactor. However, the same general trend persisted in subsequent measurement series, confirming that the observed relationship is not random. As the mole fraction of CO_2_ increases, the rate of its decomposition becomes increasingly negative, indicating an intensification of the carbon dioxide conversion process. The differences between the Step 1, Step 2, and Step 3 series are particularly visible at higher values of *y*_CO_2__ where small changes in discharge conditions may have a stronger influence on the effective reaction rate. This may result from local variations in electron density, the distribution of micro-discharges, and temporary fluctuations of the electric field in the reaction zone. Despite these deviations, the values of the fitted kinetic parameters remain similar, indicating acceptable repeatability of the process on the scale of the entire experiment. The results indicate that the proposed model may be a useful tool for comparing the plasma system’s activity under different operating conditions of the DBD reactor. Due to the limited repeatability of measurements in the DBD reactor, it was decided to fit the kinetic model separately for each measurement to capture the effect of plasma non-uniformity in the reaction zone.

**Figure 18:**
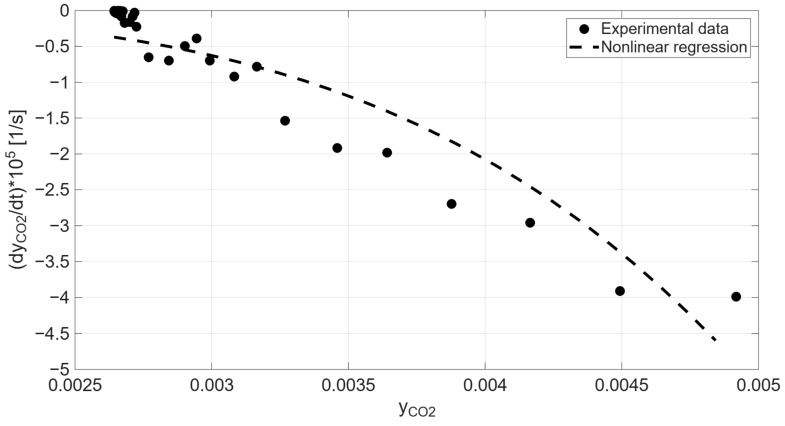
Comparison of the results of fitting the kinetic model to the experimental data-Variant A0 (Pure Kaolin, PEN = 20).

The experimental data were fitted to the proposed kinetic model using the Levenberg–Marquardt method, a commonly applied to solve non-linear least-squares problems. The calculations were performed in MATLAB using the lsqnonlin function available in the Optimisation Toolbox. The analyses were carried out using MATLAB 2025a and MATLAB 2026a, enabled verification the computational procedure in different versions of the software environment. The lsqnonlin function enabled the iterative determination of the kinetic model parameters, such as *k*_0_, *n*, and *E*_act_. During the optimisation procedure, the absolute error between the experimentally determined reaction rates and the values calculated from the kinetic model was minimised. This approach allowed deviations between the model and measured data to be directly reduced across the entire analysed range of CO_2_ mole fraction. The application of the Levenberg–Marquardt method yielded a stable fit to be obtained despite the non-linear character of the kinetic equation. The fitted parameter values were then used to assess the influence of the DBD reactor operating conditions on the rate of carbon dioxide decomposition. Based on the data presented in Figure 18, it can be observed that, for pure kaolin, the experimental results can be approximated by an equation in which the exponent of the mole fraction is equal to one. However, the analysis of the remaining fitted results showed that, due to the complexity of the plasma generation process, a three-parameter model equation provides significantly better results.

1. Kinetic Modelling and Parameter Sensitivity

The kinetic parameters (*k*_0_, *E*_act._, *n*) obtained from fitting the experimental data for variants A0, B1, and C1 are summarised in Table 5. These parameters serve as representative examples of the model’s performance, showcasing a range of fitting qualities across different experimental conditions. The selection of these specific cases allows for a clear demonstration of the model’s applicability, ranging from high-precision correlations to instances of lower statistical significance. Such variability highlights the complex and often stochastic nature of the plasma-catalytic process. Ultimately, the results indicate that while the proposed power-law model provides a satisfactory local description under specific conditions, its universality is limited by the intrinsic non-equilibrium nature of the DBD plasma.

It should be emphasised that the fitted parameters listed in Table 5 are effective empirical parameters and should not be interpreted as kinetic constants of an elementary reaction. The fitted parameter *n* should not be interpreted as a true reaction order; values of n exceeding typical kinetic limits reflect the empirical nature of the model and the strong non-linear character of the DBD process. In particular, the high values of n and the anomalous value of *k*_0_ obtained for B1 at PEN = 3.3 (Step 3) and anomalous value of *E*_act._ obtained for B1 at PEN = 1.65 (Step 1) are attributed to parameter compensation during non-linear regression under unstable discharge conditions. These anomalies are therefore not considered physically significant and indicate the limited applicability of the simple kinetic equation under selected plasma-assisted conditions.

2. Influence of the Plasma Energy Number (PEN)

A clear correlation was observed between the energy input and the quality of the fit (R^2^).

For the high energy regime (PEN = 20), all variants exhibited the highest stability and best fit consistency (R^2^ up to 0.87 for A0). In this regime, the empirical exponent *n* remained relatively low (3.2–4.3), suggesting a more predictable dependence on the CO_2_ mole fraction.

In the intermediate regime (PEN = 3.3), a significant shift in kinetic behaviour is observed. The empirical exponent n increases sharply—reaching values as high as 10.63 for pure kaolin—while the correlation coefficients begin to decline (typically ranging from 0.38 to 0.88 depending on the variant). This transition suggests that as the energy density decreases, the reaction rate becomes exponentially more sensitive to local concentration gradients.

The decrease in reliability was most apparent at the lowest power input (PEN = 1.65), where the R^2^ for the CuO-modified packing (C1) reached a minimum of 0.08. The low R^2^ values obtained for selected cases, particularly C1 at PEN = 1.65 and B1 at PEN = 1.65, demonstrate that the empirical power-law expression fails to describe the experimental data under these conditions. Therefore, the fitted parameters for these cases should not be assigned mechanistic meaning. The regression model is retained only as a local descriptive tool for cases with acceptable goodness of fit.

3. The kinetic Effect of Packing Material (Catalytic Influence)

The choice of packing material profoundly influenced the kinetic parameters. Pure kaolin (A0) showed the most consistent behaviour across measurement series, likely due to its chemically inert nature, which minimises complex surface-mediated interactions. NiO-modified Kaolin (B1) displayed the highest *E*_act._ values at PEN = 20.00, indicating a high sensitivity of the reaction rate to the energetic conditions. CuO-modified Kaolin (C1) exhibited the poorest fit quality across all conditions. The addition of CuO appears to introduce complex activation pathways and non-uniform discharge distributions, making the relationship between concentration and decomposition rate highly non-linear and difficult to generalise.

4. System Stochasticity and Model Limitations

The variability in *k*_0_, *E*_act._, and *n* between successive measurement steps (e.g., Step 1 vs. Step 2), despite identical macroscopic conditions, highlights the limited repeatability of DBD plasma generation. Factors such as non-uniform micro-discharge distribution and local electric field fluctuations necessitate separate model fitting for individual series to accurately reflect the system’s transient nature.

In summary, the results indicate that the classical kinetic model can describe part of the data with good accuracy, particularly for pure kaolin and higher PEN values. At the same time, the significant decrease in the quality of fit under selected conditions, especially for the CuO packing and low power numbers, confirms the need to apply more flexible predictive methods. Artificial neural networks (ANNs), and in particular LSTM (Long Short-Term Memory) networks, may provide an effective tool for modelling the CO_2_ decomposition process in a DBD reactor, as they allow non-linearity, variability, and the sequential character of the experimental data to be taken into account. In our case, the LSTM approach is not intended to replace the mechanistic interpretation of CO_2_ decomposition, but to provide a complementary predictive framework for highly non-linear and time-dependent DBD reactor data, for which a simple empirical kinetic equation is insufficient under selected operating conditions.

#### 3.10.3. Long Short-Term Memory Networks (LSTM Networks)

The kinetic model may be useful for local data description; however, its effectiveness decreases under conditions where strong plasma fluctuations, non-uniform micro-discharge distribution, and the additional influence of the catalytic properties of the packing are present. For this reason, the use of methods based on artificial neural networks for predicting measurement results is justified. Neural networks can learn complex, non-linear relationships without the need to impose a predefined form of the kinetic equation.

The use of LSTM networks appears particularly justified, since measurement data obtained from a DBD reactor may exhibit a sequential character and may depend not only on the current process conditions but also on the previous state of the system. The LSTM architecture enables such temporal dependencies and memory effects to be considered, which may be associated with plasma instability, changes in the packing surface, and transient fluctuations of reaction parameters. As a specialised type of recurrent neural network, the LSTM is designed for analysing sequential data and time-dependent signals. Its key feature is the ability to retain information from previous time steps to predict subsequent values.

Unlike simple recurrent neural networks, an LSTM contains dedicated gates that determine which information should be retained, discarded, or passed further through the network. Consequently, LSTM networks are well-suited to modelling dynamic processes, including concentration, temperature, pressure, and measurement-signal profiles. In the present study, the LSTM network was used to approximate the outlet CO_2_ mole fraction from the reactor as a function of time and power number. The model was developed using the Deep Learning Toolbox in MATLAB, which enabled the definition of the network architecture, the training procedure, and the prediction of output values.

The specific network configuration utilised a sequence input layer for the normalised time and PEN features, a single hidden LSTM layer operating in sequence-output mode, and a fully connected layer coupled with a regression layer for continuous response prediction. Training was executed using the Adam optimiser by minimising the mean squared error (MSE) loss function over 4000 epochs. To ensure full reproducibility of the machine learning workflow, the comprehensive specifications of the network architecture, data preprocessing steps, and training hyperparameters are provided in Supplementary Material S4.1, Table S3.

**Figure 19:**
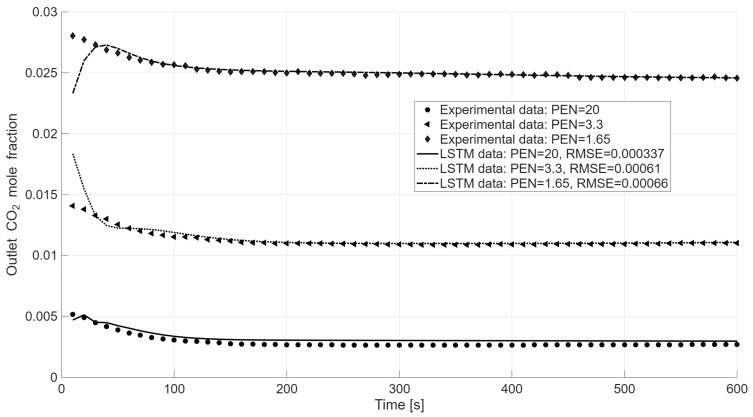
Comparison of experimental and LSTM-predicted profiles of the outlet CO_2_ mole fraction as a function of time for different Plasma Energy Number (PEN) values for variant A0.

As presented in Figure 19, for an exemplary variant A0, the LSTM network accurately reproduces the experimental profiles of the outlet CO_2_ mole fraction across all three power-number values. The best fit was obtained for PEN = 20, with an RMSE of 0.000337; the model correctly captured both the initial decrease and the steady-state region. For PEN = 3.3, the model also followed the temporal trend well, although the error was slightly higher (RMSE = 0.00061). The highest error was observed for PEN = 1.65 (RMSE = 0.00066), primarily due to the more challenging representation of the initial maximum in the profile.

In all cases, the network correctly predicts the system’s tendency to approach steady-state values over longer process times. The largest discrepancies between the experimental data and the LSTM predictions occur at the beginning of the profiles, where the CO_2_ concentration changes most dynamically. After this transient stage, the agreement is excellent, with the prediction curves almost overlapping the experimental data points. The obtained RMSE values confirm that the LSTM network effectively approximates the dependence of the CO_2_ mole fraction on both time and power number.

The LSTM model was used in this work as a preliminary data-driven predictive tool for time-dependent DBD reactor data. However, the present study does not include a systematic comparison with alternative time-series models, such as ARIMA (AutoRegressive Integrated Moving Average), feed-forward neural networks or simple RNNs; therefore, such a comparison should be regarded as an important direction for future work.

## 4. Conclusions

In this study, the performance and dynamic behaviour of kaolin-based catalysts modified with Ni, Ni–Ce, and Fe–Cu oxides were systematically investigated in a Dielectric Barrier Discharge (DBD) reactor for CO_2_ conversion. The results provide new insights into plasma–material interactions and highlight the critical role of both the chemical composition and the physical properties of the catalytic systems.

The unmodified kaolin support (A0) exhibited relatively high CO_2_ conversion, demonstrating its active role as a dielectric material that influences micro-discharge formation. This confirms that support cannot be considered inert in plasma-catalytic processes. Among the investigated materials, Ni-based catalysts demonstrated the highest activity, with CO_2_ conversion reaching approximately 53% for the sample calcined at 500 °C. The observed enhancement with increasing calcination temperature suggests that the physicochemical properties of the Ni phase—such as crystallinity and dispersion—significantly dictate plasma-catalytic performance.

In contrast, the incorporation of CeO_2_ led to a pronounced decrease in conversion and introduced significant temporal instability. This suggests that CeO_2_ may promote the recombination of reactive oxygen species or alter the dielectric properties, thereby reducing plasma efficiency. Similarly, Fe–Cu-based systems exhibited low and highly variable activity, indicating the absence of a stable active phase under the investigated DBD conditions.

Time-resolved analysis revealed that steady-state measurements alone are insufficient to fully characterise catalyst performance. While Ni-based systems exhibited stable operation, catalysts containing CeO_2_ and Fe–Cu oxides showed distinct transient behaviour, characterised by initial activity followed by gradual deactivation. These findings highlight the dynamic nature of plasma-catalytic systems and the need for temporal analysis to evaluate stability. Finally, the observed instabilities and deactivation in certain systems suggest that alternative reactor configurations should be explored. Future research could benefit from investigating post-plasma catalysis (PPC) layouts, in which the discharge zone is physically separated from the catalytic bed. Such a two-stage configuration may mitigate adverse plasma–material interactions, such as surface restructuring or deactivation by short-lived reactive species, thereby enhancing the long-term stability and selectivity of the process.

Overall, CO_2_ conversion in DBD reactors is governed by a complex interplay between plasma chemistry and material properties. Consequently, catalyst design must account for both chemical activity and dielectric influence on discharge behaviour. Furthermore, the generated time-series data provide a valuable foundation for the application of machine learning methods to predict catalyst performance and stability.

## Figures and Tables

**Table 1:** Summary of the investigated kaolin-based catalysts, including their nominal compositions, synthesis strategies, and anticipated surface characteristics.

Variant	Composition	Impregnation Method	Structure
A0	Pure Kaolin	Support only	Bare aluminosilicate surface
B1	Kaolin + 10 wt.% NiO	Wet impregnation	Isolated NiO clusters on kaolin
B1′	Kaolin + 10 wt.% NiO	Wet impregnation (500 °C)	Enhanced NiO-kaolin interaction
B2	Kaolin + 10 wt.% NiO + 10 wt.% CeO_2_	Co-impregnation	Intimate NiO-CeO_2_ contact
B3	Kaolin + 10 wt.% NiO + 10 wt. % CeO_2_	Sequential	Layered structure (NiO on CeO_2_)
C1	Kaolin + 10 wt.% CuO	Wet impregnation	Dispersed CuO phases
C2	Kaolin + 10 wt.% Fe_2_O_3_	Wet impregnation	Dispersed Fe_2_O_3_ phases
C3	Kaolin + 5 wt.% CuO + 5 wt.% Fe_2_O_3_	Co-impregnation	Potential CuFe_2_O_4_ spinel formation
C4	Kaolin + 5 wt.% CuO + 5 wt.% Fe_2_O_3_	Sequential	Fe-oxide core with Cu-oxide shell
C5	Kaolin + 5 wt.% CuO + 5 wt.% Fe_2_O_3_ + 5 wt.% CeO_2_	Mixed impregnation	Triple-phase synergy (Cu-Fe-Ce)

**Table 2:** Crystallite sizes and structural parameters of the active phases determined by Rietveld refinement.

Sample	Identified Phase	Space Group	Lattice Parameters [Å]	Crystallite Size [nm]	ICDD Card No.
B1	NiO CeO_2_	*Fm*3^−^*m* *Fm*3^−^*m*	a = 4.180 a = 5.412	6.3 51.7	04-001-9373 00-064-0737
B1′	NiO	*Fm*3^−^*m*	a = 4.179	12.9	04-001-9373
B2	NiO CeO_2_	*Fm*3^−^*m* *Fm*3^−^*m*	a = 4.179 a = 5.407	10.4 3.4	04-001-9373 00-064-0737
B3	NiO CeO_2_	*Fm*3^−^*m* *Fm*3^−^*m*	a = 4.179 a = 5.414	11.4 4.5	04-001-9373 00-064-0737
C1	CuO	*C*1*c*1	a = 4.686, b = 3.439, c = 5.127	67.9	04-007-0518
C2	Fe_2_O_3_	*R*3^−^*c*	a = 9.612, b = 5.033, c = 13.741	10.7	00-033-0664
C4	CuO	*C*1*c*1	a = 4.688, b = 3.432, c = 5.125	81.0	04-007-0518

**Table 3:** Summary of the investigated kaolin-based catalysts: nominal compositions, synthesis strategies, and estimated oxide contents derived from EDXRF analysis.

Variant	Composition	Impregnation Method	Calculated Oxide Content Based on EDXRF (wt.%) ^1^
A0	Pure Kaolin	Support only	Fe_2_O_3_: 1.17 ^2^
B1	Kaolin + 10 wt.% NiO	Wet impregnation	NiO: 13.10, Fe_2_O_3_: 0.99 ^2^
B1′	Kaolin + 10 wt.% NiO	Wet impregnation (500 °C)	NiO: 12.68
B2	Kaolin + 10 wt.% NiO + 10 wt.% CeO_2_	Co-impregnation	NiO: 10.08, CeO_2_: 10.24, Fe_2_O_3_: 1.20 ^2^
B3	Kaolin + 10 wt.% NiO + 10 wt. % CeO_2_	Sequential	NiO: 11.61, CeO_2_: 17.09, Fe_2_O_3_: 1.16 ^2^
C1	Kaolin + 10 wt.% CuO	Wet impregnation	CuO: 11.78, Fe_2_O_3_: 0.97 ^2^
C2	Kaolin + 10 wt.% Fe_2_O_3_	Wet impregnation	Fe_2_O_3_: 14.45
C3	Kaolin + 5 wt.% CuO + 5 wt.% Fe_2_O_3_	Co-impregnation	CuO: 7.65, Fe_2_O_3_: 6.44
C4	Kaolin + 5 wt.% CuO + 5 wt.% Fe_2_O_3_	Sequential	CuO: 7.98, Fe_2_O_3_: 6.62
C5	Kaolin + 5 wt.% CuO + 5 wt.% Fe_2_O_3_ + 5 wt.% CeO_2_	Mixed impregnation	CuO: 7.42, Fe_2_O_3_: 6.08, CeO_2_: 6.34

^1^ Oxide content calculated based on the elemental composition determined by EDXRF, assuming stoichiometric oxidation states (NiO, CeO_2_, CuO, Fe_2_O_3_). ^2^ Indicates residual Fe_2_O_3_ naturally occurring in the kaolin support.

**Table 4:** Surface atomic composition (at.%) of kaolin-based catalysts before and after plasma-catalytic CO_2_ decomposition determined by Energy-Dispersive X-ray Spectroscopy (EDS).

Variant	State	C	O	Al	Si	Active Phase (at.%)	Others
A0	Fresh	—	63.91	21.16	13.94	—	K: 1.00
	Spent	7.65	57.47	18.07	14.94	—	K: 0.73, Ca: 0.60
B1	Fresh	—	53.70	16.15	11.33	Ni: 18.82	—
	Spent	9.80	52.49	14.09	11.62	Ni: 11.42, Fe: 0.58	—
B1’	Fresh Spent	— 9.99	54.56 46.66	19.23 14.35	12.89 10.36	Ni: 12.51 Ni: 18.62	K: 0.80
B2	Fresh	—	44.98	12.98	11.07	Ni: 15.80, Ce: 15.17	—
	Spent	10.45	45.39	10.10	8.38	Ni: 13.14, Ce: 12.54	—
B3	Fresh	—	48.54	15.31	10.46	Ni: 18.81, Ce: 6.89	—
	Spent	10.28	44.14	10.56	8.78	Ni: 23.08, Ce: 3.16	—
C1	Fresh	—	57.53	21.57	14.10	Cu: 5.88	K: 0.92
	Spent	8.64	39.74	13.14	8.73	Cu: 29.76	—
C2	Fresh	—	48.12	16.22	9.79	Fe: 25.87	—
	Spent	10.29	50.18	8.36	6.10	Fe: 25.08	—
C3	Fresh	—	52.00	18.33	11.41	Fe: 8.27, Cu: 9.98	—
	Spent	14.10	24.02	5.10	3.57	Fe: 35.95, Cu: 17.27	—
C4	Fresh	—	55.91	17.79	11.80	Fe: 6.02, Cu: 8.47	—
	Spent	10.89	51.18	15.20	11.22	Fe: 4.39, Cu: 7.13	—
C5	Fresh	—	48.19	16.43	10.24	Fe: 12.97, Cu: 9.24, Ce: 2.93	—
	Spent	9.94	42.88	10.27	07.09	Fe: 20.00, Cu: 7.95, Ce: 1.88	—

**Table 5:** Comparison of the fitting results for three different types of DBD reactor packing.

Variant	Composition	Step No.	PEN	*k*_0_	*E*_act._	*n*	R^2^
A0	Pure Kaolin	1	20.00	80.83	155.67	4.16	0.87
A0	Pure Kaolin	2	20.00	73.18	109.81	3.72	0.86
A0	Pure Kaolin	3	20.00	87.92	144.21	4.04	0.86
A0	Pure Kaolin	1	3.30	29.55	106.98	10.63	0.83
A0	Pure Kaolin	2	3.30	31.53	102.08	10.31	0.83
A0	Pure Kaolin	3	3.30	27.23	80.86	8.80	0.77
A0	Pure Kaolin	1	1.65	19.32	69.69	15.45	0.65
A0	Pure Kaolin	2	1.65	18.49	72.85	15.91	0.73
A0	Pure Kaolin	3	1.65	18.50	68.04	15.18	0.73
B1	Kaolin + 10 wt.% NiO	1	20.00	93.09	163.15	4.25	0.85
B1	Kaolin + 10 wt.% NiO	2	20.00	83.49	132.80	3.95	0.8
B1	Kaolin + 10 wt.% NiO	3	20.00	66.17	92.06	3.54	0.81
B1	Kaolin + 10 wt.% NiO	1	3.30	36.40	125.40	12.08	0.76
B1	Kaolin + 10 wt.% NiO	2	3.30	34.81	113.63	11.26	0.68
B1	Kaolin + 10 wt.% NiO	3	3.30	492.93 ^1^	114.59	11.97	0.88
B1	Kaolin + 10 wt.% NiO	1	1.65	9.81	0.04 ^1^	3.45	0.04 ^2^
B1	Kaolin + 10 wt.% NiO	2	1.65	26.14	101.53	20.89	0.66
B1	Kaolin + 10 wt.% NiO	3	1.65	59.37	121.03	24.45	0.82
C1	Kaolin + 10 wt.% CuO	1	20.00	52.48	57.22	3.18	0.75
C1	Kaolin + 10 wt.% CuO	2	20.00	62.5	81.12	3.43	0.65
C1	Kaolin + 10 wt.% CuO	3	20.00	51.86	57.94	3.21	0.47
C1	Kaolin + 10 wt.% CuO	1	3.30	21.91	48.47	6.64	0.42
C1	Kaolin + 10 wt.% CuO	2	3.30	28.37	84.85	9.23	0.41
C1	Kaolin + 10 wt.% CuO	3	3.30	31.59	105.50	10.70	0.38
C1	Kaolin + 10 wt.% CuO	1	1.65	19.15	55.95	13.27	0.26
C1	Kaolin + 10 wt.% CuO	2	1.65	14.49	20.25	7.25	0.08 ^2^
C1	Kaolin + 10 wt.% CuO	3	1.65	29.02	64.03	14.81	0.33

^1^ Indicates anomalous values. ^2^ Indicates poor goodness of fit.

## Data Availability

The original contributions presented in this study are included in the article/Supplementary Material. Further inquiries can be directed to the corresponding authors.

## Supplementary Materials

The following supporting information can be downloaded at: https://www.mdpi.com/article/10.3390/ma19132747/s1. Figure S1: Representative Q–U Lissajous loop for the plasma–catalytic CO_2_ decomposition; the experimental data is overlaid with an idealized trapezoidal profile used to determine the energy dissipated per cycle; Figure S2: SEM-EDS elemental mapping of the spent A0 catalyst surface showing the distribution of: (a) nickel (Ni), (b) iron (Fe), (c) carbon (C), and (d) all elements superimposed (magnification 250×); Figure S3: SEM-EDS elemental mapping of the spent B1 catalyst surface showing the distribution of: (a) nickel (Ni, magnification 180×), (b) iron (Fe, magnification 180×), (c) carbon (C, magnification 250×), and (d) superimposed Fe and Ni elements (magnification 180×); Figure S4: SEM-EDS elemental mapping of the spent B1’ catalyst surface showing the distribution of: (a) nickel (Ni), (b) iron (Fe), (c) carbon (C), and (d) all elements superimposed (magnification 300×); Figure S5: SEM-EDS elemental mapping of the spent B2 catalyst surface showing the distribution of: (a) nickel (Ni), (b) iron (Fe), (c) carbon (C), and (d) all elements superimposed (magnification 300×); Figure S6: SEM-EDS elemental mapping of the spent B3 catalyst surface showing the distribution of: (a) nickel (Ni), (b) iron (Fe), (c) carbon (C), and (d) cerium (Ce) (magnification 300×); Figure S7: SEM-EDS elemental mapping of the spent B3 catalyst surface showing the distribution of: nickel (Ni), iron (Fe), carbon (C), and cerium (Ce) (magnification 300×); Figure S8: SEM-EDS elemental mapping of the spent C1 catalyst surface showing the distribution of: (a) nickel (Ni), (b) iron (Fe), (c) carbon (C), and (d) cerium (Ce) (magnification 300×); Figure S9: SEM-EDS elemental mapping of the spent C1 catalyst surface showing the distribution of: nickel (Ni), iron (Fe), carbon (C), and cerium (Ce) (magnification 300×); Figure S10: SEM-EDS elemental mapping of the spent C2 catalyst surface showing the distribution of: (a) nickel (Ni), (b) iron (Fe), (c) copper (C), and (d) cerium (Ce) (magnification 300×); Figure S11: SEM-EDS elemental mapping of the spent C2 catalyst surface showing the distribution of: (a) carbon (C), and (b) all elements superimposed (magnification 300×); Figure S12: SEM-EDS elemental mapping of the spent C3 catalyst surface showing the distribution of: (a) copper (Cu, magnification 180×), (b) iron (Fe, magnification 180×), (c) carbon (C, magnification 300×), and (d) superimposed Fe and Cu elements (magnification 180×); Figure S13: SEM-EDS elemental mapping of the spent C4 catalyst surface showing the distribution of: (a) copper (Cu), (b) iron (Fe), (c) carbon (C), and (d) all elements superimposed (magnification 180×); Figure S14: SEM-EDS elemental mapping of the spent C5 catalyst surface showing the distribution of: (a) copper (Cu), (b) iron (Fe), (c) cerium (Ce), and (d) superimposed Fe, Cu, and Ce elements (magnification 180×); Figure S15: SEM-EDS elemental mapping showing the surface distribution of carbon (C) on the spent C5 catalyst (magnification 300×; Table S1: Elemental composition (wt.%) of the pristine kaolin and metal-modified catalysts determined by EDXRF; Table S2: Microstructural and coverage characteristics of element-rich regions determined from semi-quantitative SEM-EDS map analysis; Table S3: Technical specifications, data preprocessing steps, and training hyperparameters of the developed LSTM network architecture.

## Abbreviations

The following abbreviations are used in this manuscript:

NTP	Non-Thermal Plasma
DBD	Dielectric Barrier Discharge
PEN	Plasma Energy Number
XRD	X-ray Diffraction
EDXRF	Energy-Dispersive X-ray Fluorescence Spectroscopy
SEM-EDS	Scanning Electron Microscopy with Energy Dispersive X-ray Spectroscopy
DBD-PB	Dielectric Barrier Discharge Packed-Bed configuration
GND	Ground
SEI	Specific Energy Input
ICDD	International Centre for Diffraction Data
VT	Vibrational–Translational
LSTM	Long Short-Term Memory networks
ANNs	Artificial Neural Networks
ARIMA	AutoRegressive Integrated Moving Average
