# Supplementary material for: Temporal Evolution of CO2 Conversion over Kaolin-Supported Ni, Ni–Ce and Fe–Cu Catalysts Under Dielectric Barrier Discharge Conditions
Source: Materials (Basel). 2026 Jun 26;19(13):2747. doi: 10.3390/ma19132747 (PMC13362766; doi:10.3390/ma19132747)
Supplement: Supplementary file 1 [file materials-19-02747-s001.zip › materials-4335850-supplementary.pdf]

materials

MDPI

Article

Temporal Evolution of CO_2 Conversion over Kaolin-Supported Ni, Ni–Ce and Fe–Cu Catalysts under Dielectric Barrier Discharge Conditions

Agata Dorosz [1,*], Michał Lewak [1], Katarzyna Jabłczyńska [1], Marta Mazurkiewicz-Pawlicka [1], Jakub Trzciński [2], Krzysztof Zaraska [3], Piotr Maćków [3], Jakub Jaworski [1] and Arkadiusz Moskal [1,*]

1 Faculty of Chemical and Process Engineering, Warsaw University of Technology, ul. Waryńskiego 1, 00-645 Warsaw, Poland; michal.lewak@pw.edu.pl (M.L.); katarzyna.jablczynska@pw.edu.pl (K.J.); marta.pawlicka@pw.edu.pl (M.M.-P.); jakub.jaworski10.stud@pw.edu.pl (J.J.)
2 Centre for Advanced Materials and Technologies CEZAMAT, Warsaw University of Technology, Poleczki 19, 02-822 Warsaw, Poland; jakub.trzcinski@pw.edu.pl
3 Łukasiewicz Research Network—Institute of Microelectronics and Photonics, Krakow Division, ul. Zabłocie 39, 30-701 Krakow, Poland; krzysztof.zaraska@imif.lukasiewicz.gov.pl (K.Z.); piotr.mackow@imif.lukasiewicz.gov.pl (P.M.)
* Correspondence: agata.dorosz@pw.edu.pl (A.D.); arkadiusz.moskal@pw.edu.pl (A.M.)

Appendix A

Appendix A.1

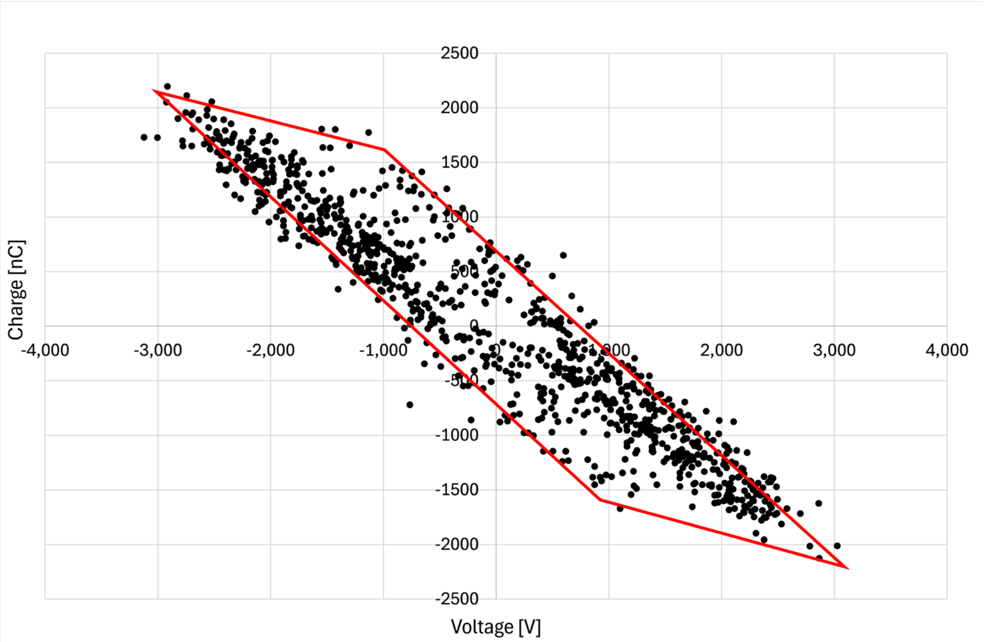

Figure S1. Representative *Q*–*U* Lissajous loop for the plasma–catalytic CO_2 decomposition; the experimental data is overlaid with an idealized trapezoidal profile used to determine the energy dissipated per cycle.

Appendix B

Appendix B.1

Table S1. Elemental composition (wt.%) of the pristine kaolin and metal-modified catalysts determined by EDXRF.

Academic Editor: Mirosław Dors

Received: date
Revised: date
Accepted: date
Published: date

Copyright: © 2026 by the authors. Submitted for possible open access publication under the terms and conditions of the Creative Commons Attribution (CC BY) license.

Element	A0 Pure Kaolin	B1<br>10 wt.%<br>NiO 400 °C	B1′<br>10 wt.%<br>NiO 500 °C	B2<br>10 wt.% NiO<br>+ 5 wt.% CeO_2<br>co-impregnation	B3<br>10 wt.% NiO<br>+ 5 wt.% CeO_2<br>sequential<br>impregnation
Al	15.921 %	17.898 %	18.745 %	20.100 %	18.954 %
Si	23.594 %	22.575 %	23.908 %	23.511 %	21.179 %
K	2.289 %	1.945 %	1.991 %	1.921 %	1.749 %
Fe	0.815 %	0.693 %	--	0.837 %	0.809 %
Cu	--	--	--	--	
Ce	--	--	--	8.334 %	13.914 %
Ni	--	9.966 %	10.292 %	7.921 %	9.120 %
**Element**	**C1<br>10 wt.% CuO**	**C2<br>10 wt.% Fe_2O_3**	**C3<br>5 wt.% CuO<br>+ 5 wt. % Fe_2O_3<br>co-impregnation**	**C4<br>5 wt.% CuO<br>+ 5 wt. % Fe_2O_3<br>sequential<br>impregnation**	**C5<br>5 wt.% CuO<br>+ 5 wt. % Fe_2O_3<br>+ 5 wt.% CeO_2**
Al	19.808 %	18.278 %	19.139%	19.191 %	19.561 %
Si	24.316 %	23.623%	24.155%	23.929 %	23.018 %
K	1.979 %	1.988 %	2.020 %	1.980 %	1.914 %
Fe	0.679 %	10.924 %	5.322 %	5.444 %	5.068 %
Cu	9.410 %	--	6.112 %	6.377 %	5.924 %
Ce	--	--	--	--	5.161 %
Ni	--	--	--	--	--

Appendix C

Appendix C.1

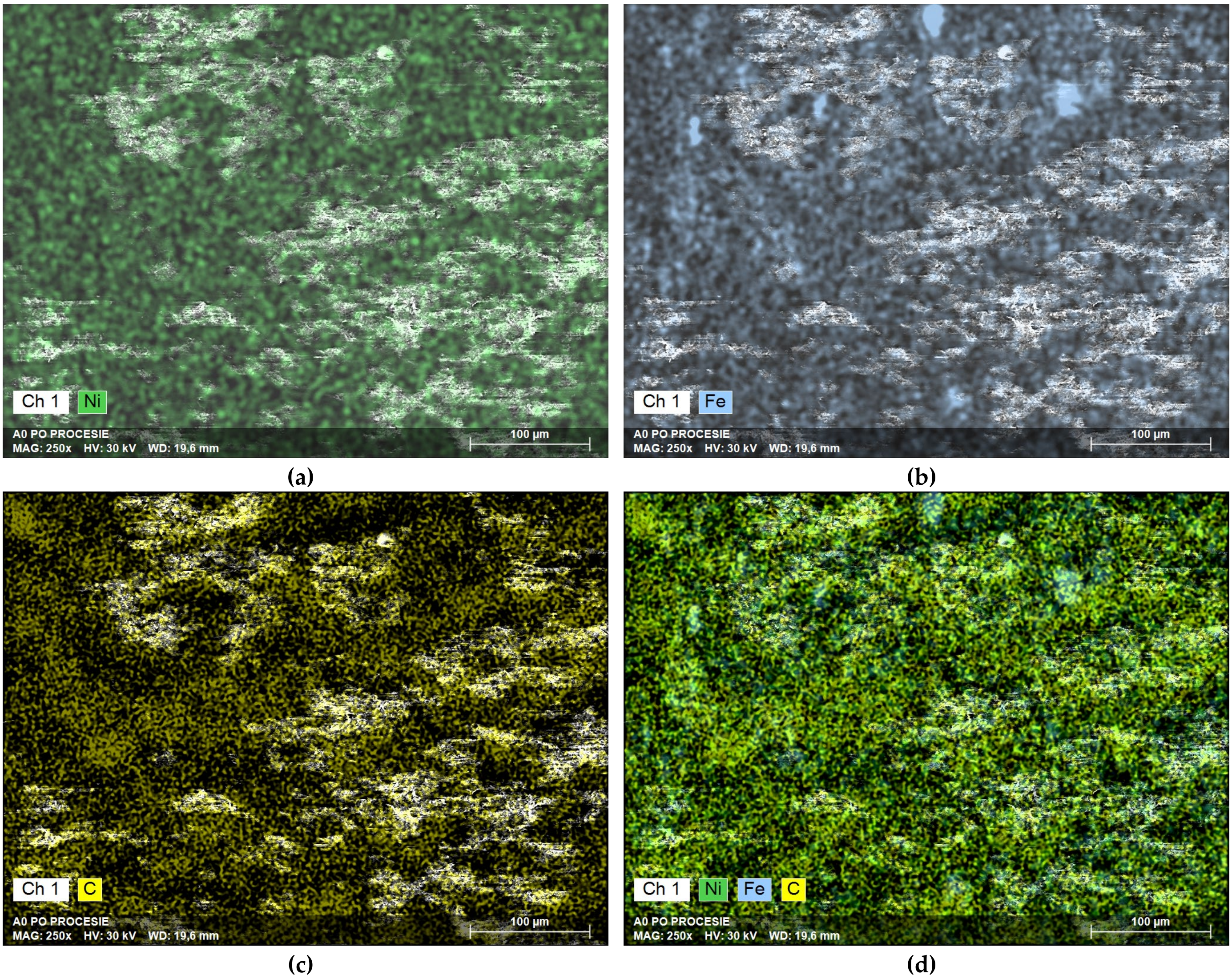

Figure S2. SEM-EDS elemental mapping of the spent A0 catalyst surface showing the distribution of: (a) nickel (Ni), (b) iron (Fe), (c) carbon (C), and (d) all elements superimposed (magnification 250×).

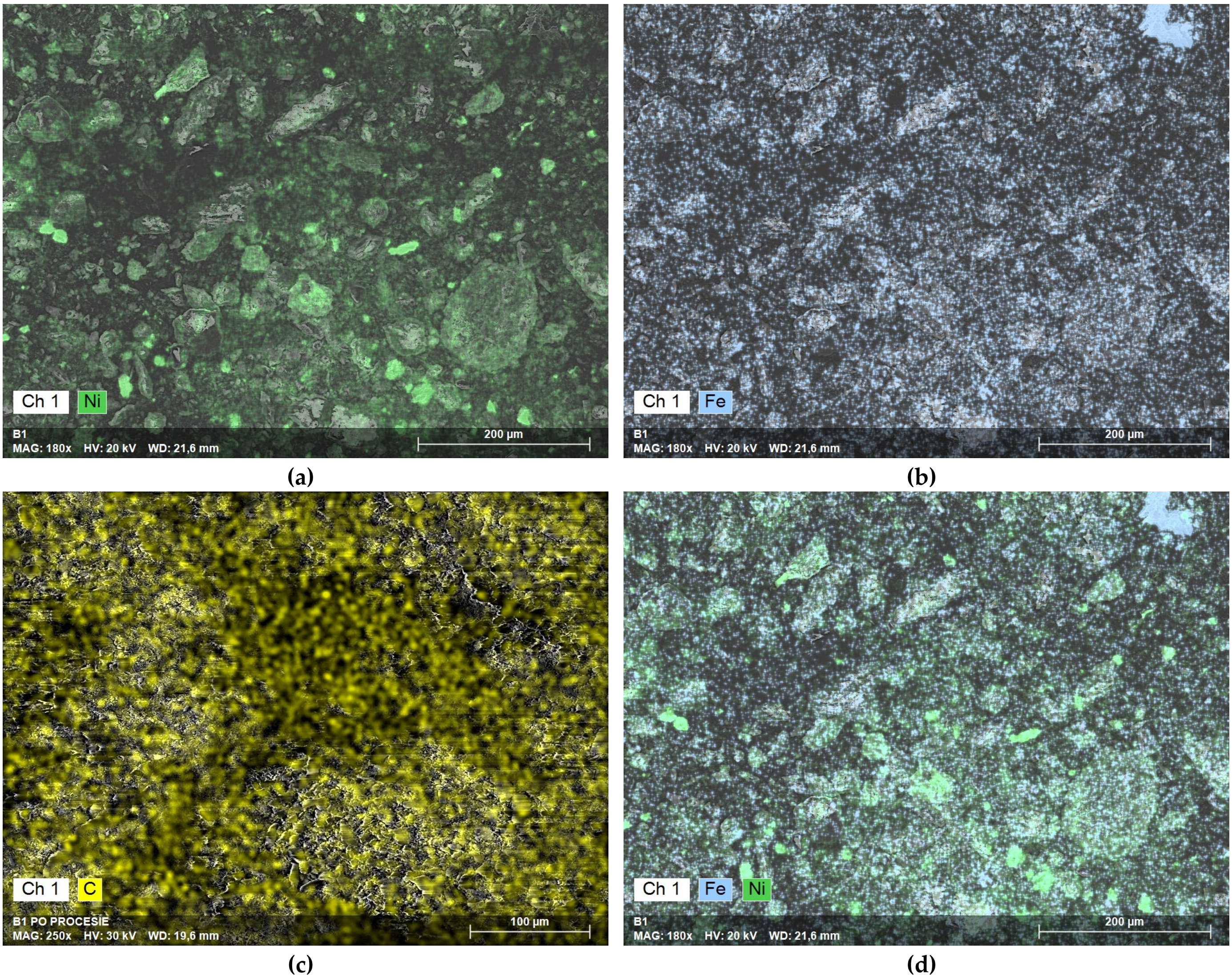

Figure S3. SEM-EDS elemental mapping of the spent B1 catalyst surface showing the distribution of: (a) nickel (Ni, magnification 180×), (b) iron (Fe, magnification 180×), (c) carbon (C, magnification 250×), and (d) superimposed Fe and Ni elements (magnification 180×).

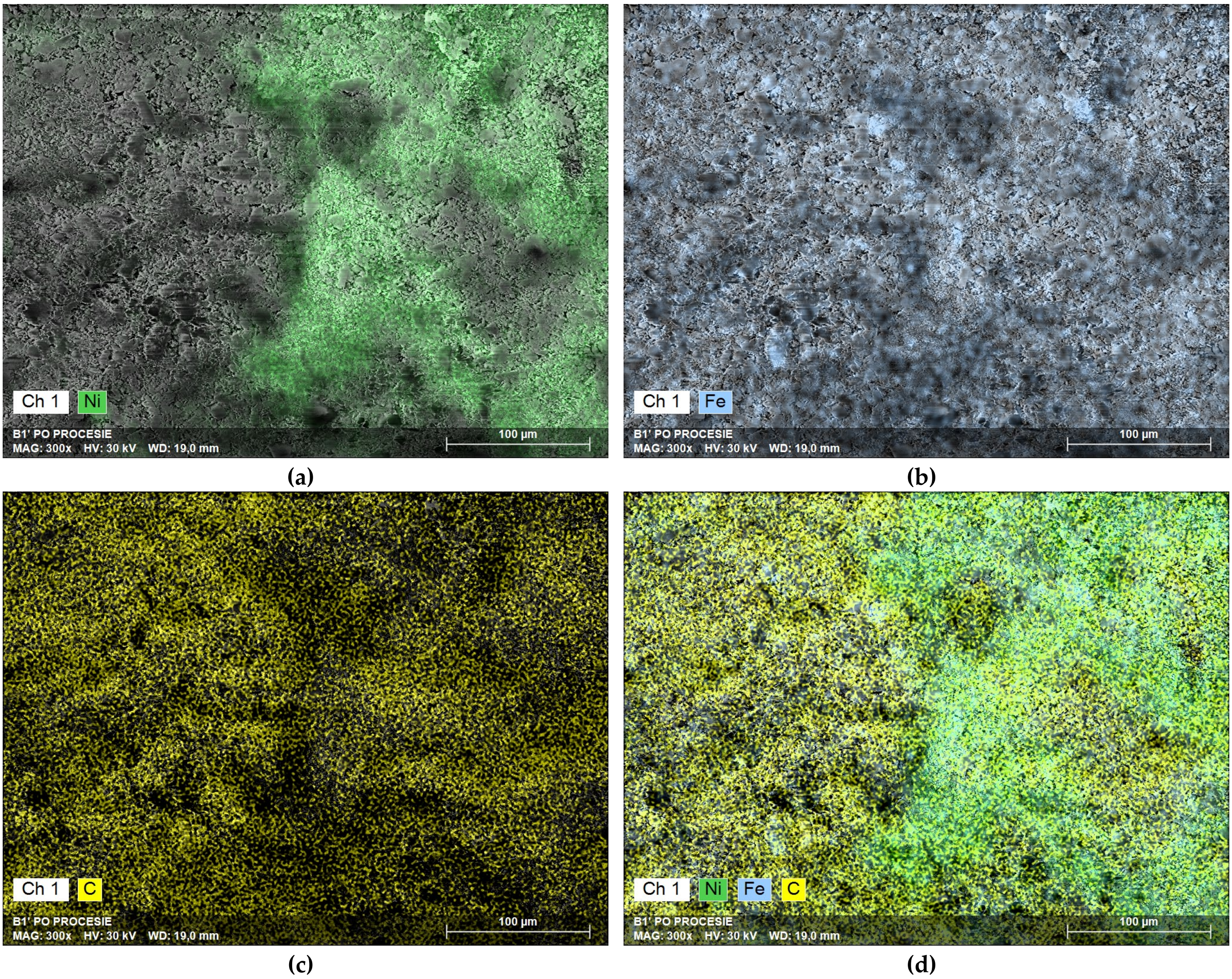

Figure S4. SEM-EDS elemental mapping of the spent B1′ catalyst surface showing the distribution of: (a) nickel (Ni), (b) iron (Fe), (c) carbon (C), and (d) all elements superimposed (magnification 300×).

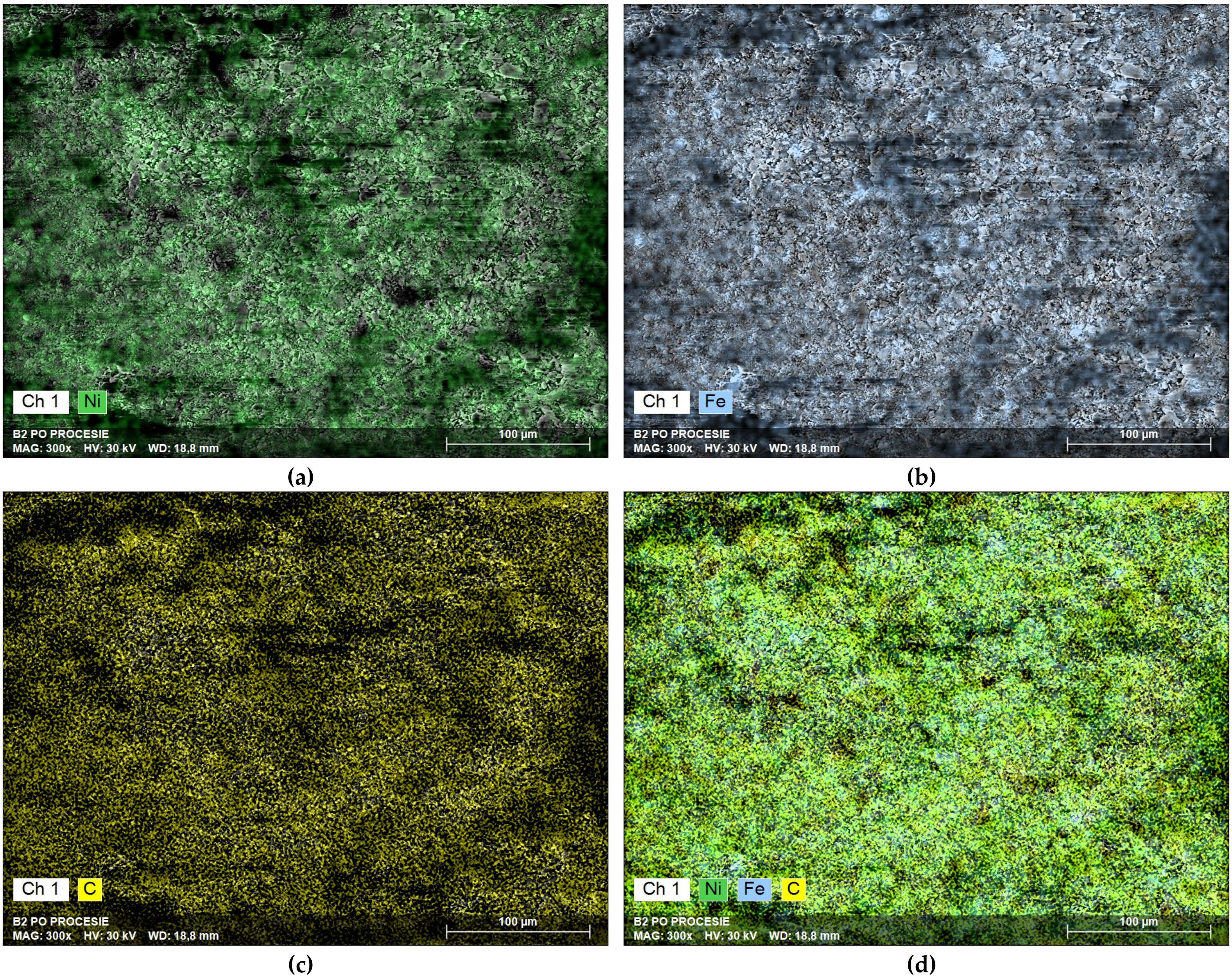

Figure S5. SEM-EDS elemental mapping of the spent B2 catalyst surface showing the distribution of: (a) nickel (Ni), (b) iron (Fe), (c) carbon (C), and (d) all elements superimposed (magnification 300×).

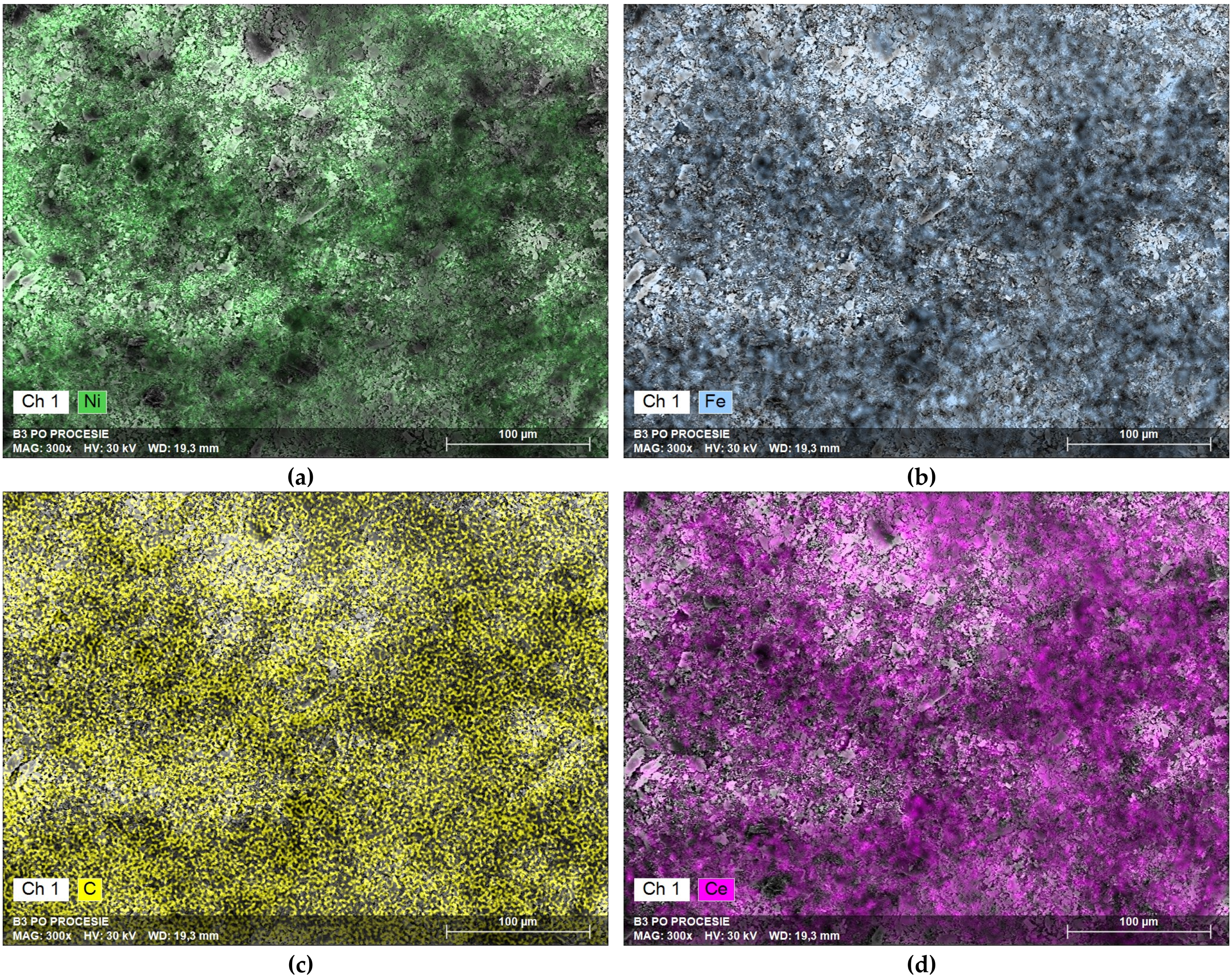

Figure S6. SEM-EDS elemental mapping of the spent B3 catalyst surface showing the distribution of: (a) nickel (Ni), (b) iron (Fe), (c) carbon (C), and (d) cerium (Ce) (magnification 300×).

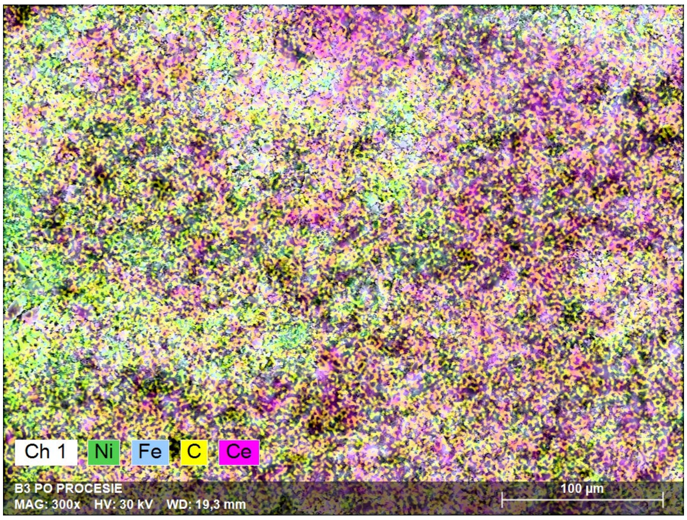

Figure S7. SEM-EDS elemental mapping of the spent B3 catalyst surface showing the distribution of: nickel (Ni), iron (Fe), carbon (C), and cerium (Ce) (magnification 300×).

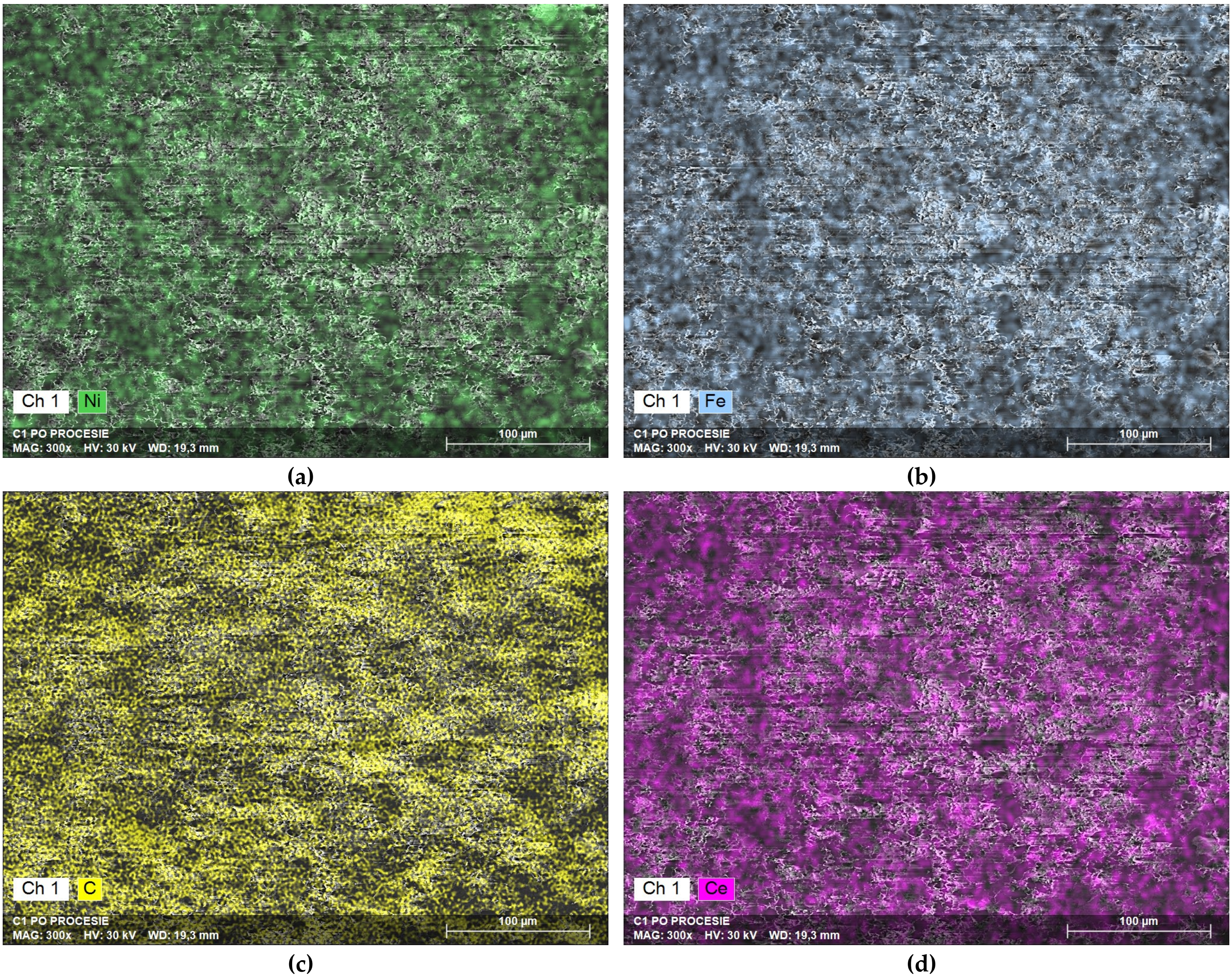

Figure S8. SEM-EDS elemental mapping of the spent C1 catalyst surface showing the distribution of: (a) nickel (Ni), (b) iron (Fe), (c) carbon (C), and (d) cerium (Ce) (magnification 300×).

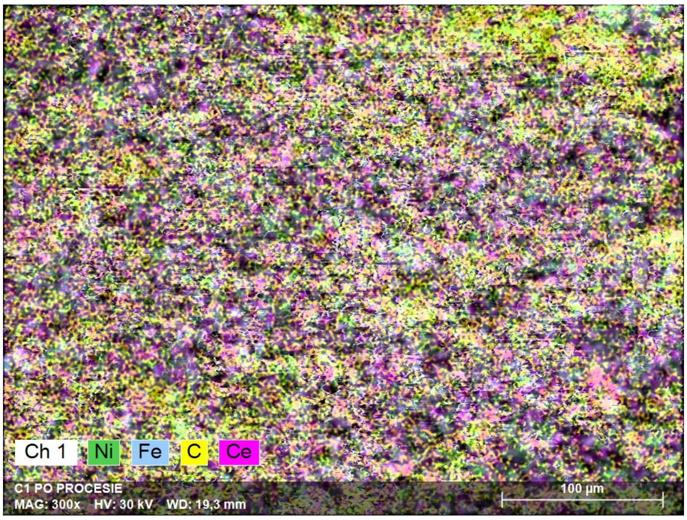

Figure S9. SEM-EDS elemental mapping of the spent C1 catalyst surface showing the distribution of: nickel (Ni), iron (Fe), carbon (C), and cerium (Ce) (magnification 300×).

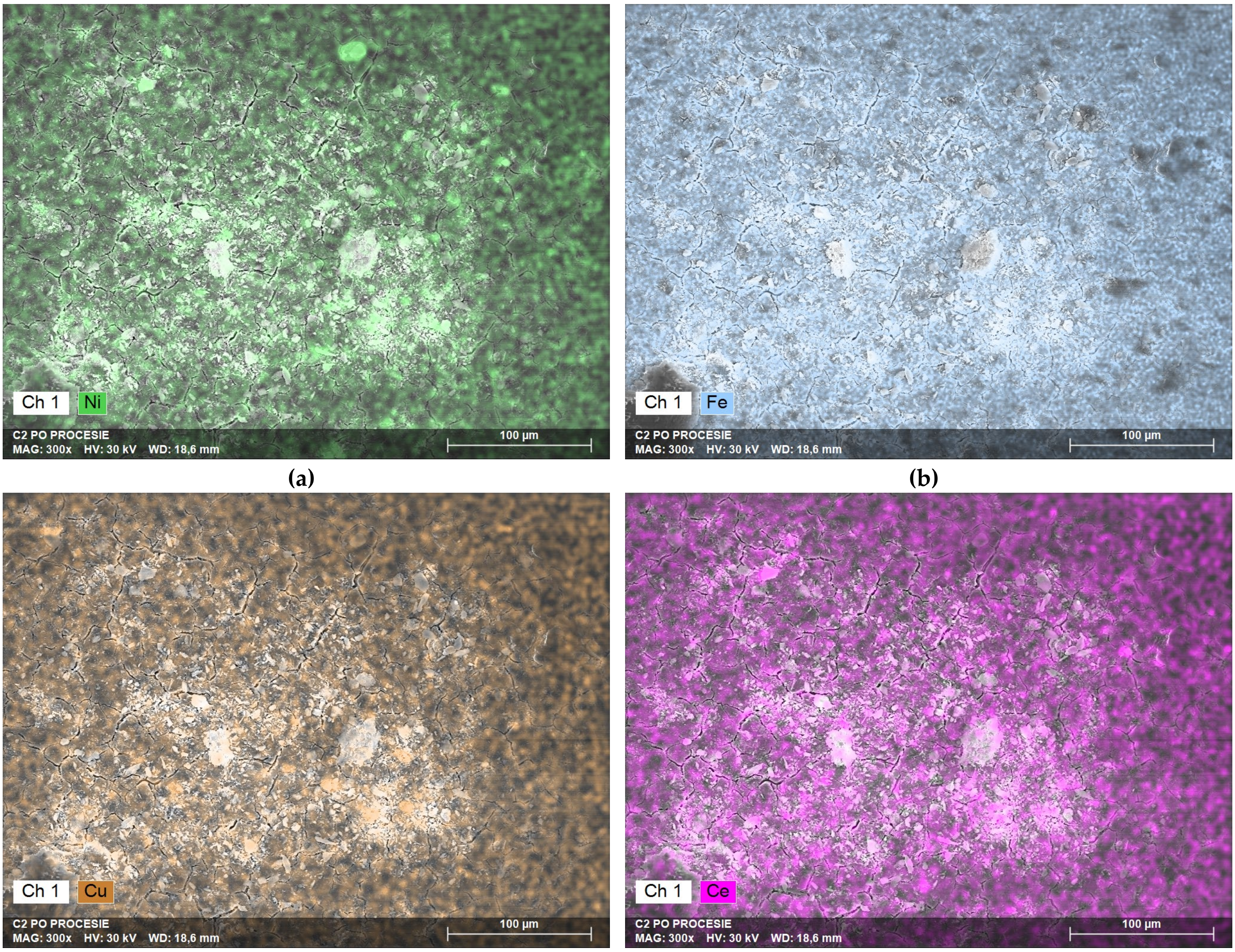

(a) **(b)**

(c) **(d)**

Figure S10. SEM-EDS elemental mapping of the spent C2 catalyst surface showing the distribution of: (a) nickel (Ni), (b) iron (Fe), (c) copper (C), and (d) cerium (Ce) (magnification 300×).

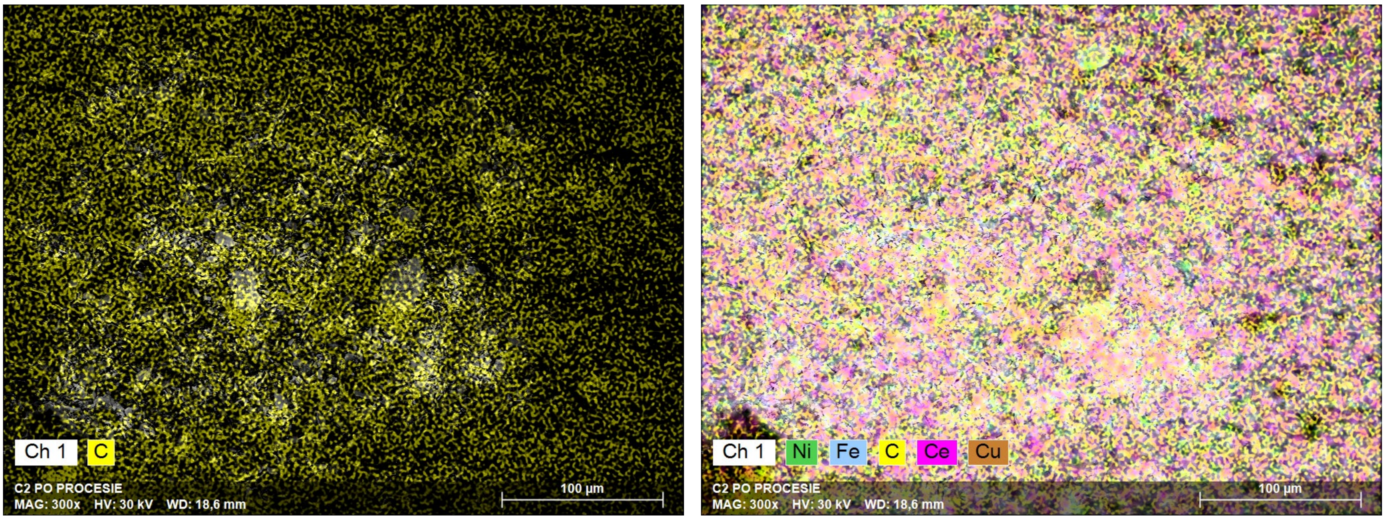

(a) **(b)**

Figure S11. SEM-EDS elemental mapping of the spent C2 catalyst surface showing the distribution of: (a) carbon (C), and (b) all elements superimposed (magnification 300×).

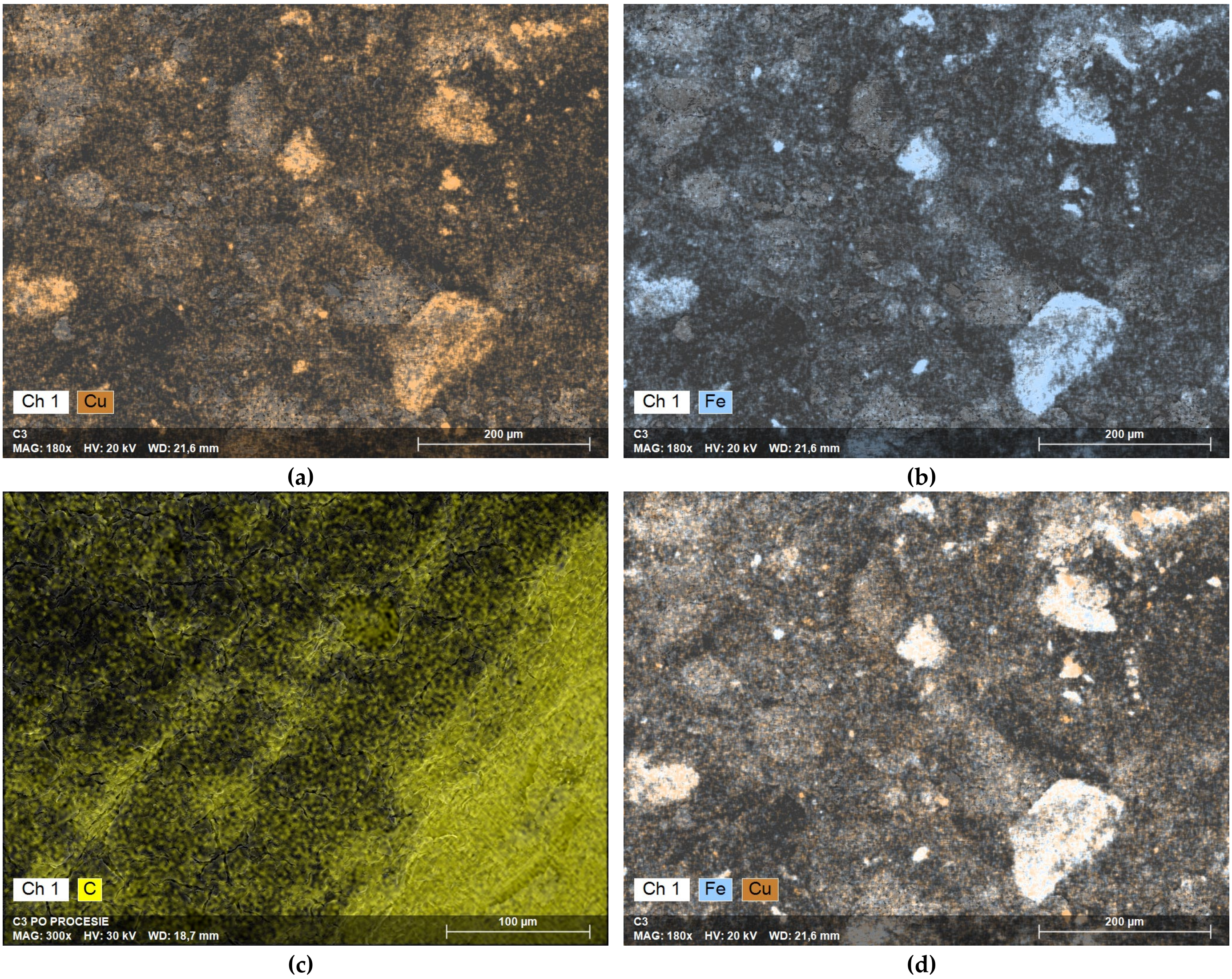

Figure S12. SEM-EDS elemental mapping of the spent C3 catalyst surface showing the distribution of: (a) copper (Cu, magnification 180×), (b) iron (Fe, magnification 180×), (c) carbon (C, magnification 300×), and (d) superimposed Fe and Cu elements (magnification 180×).

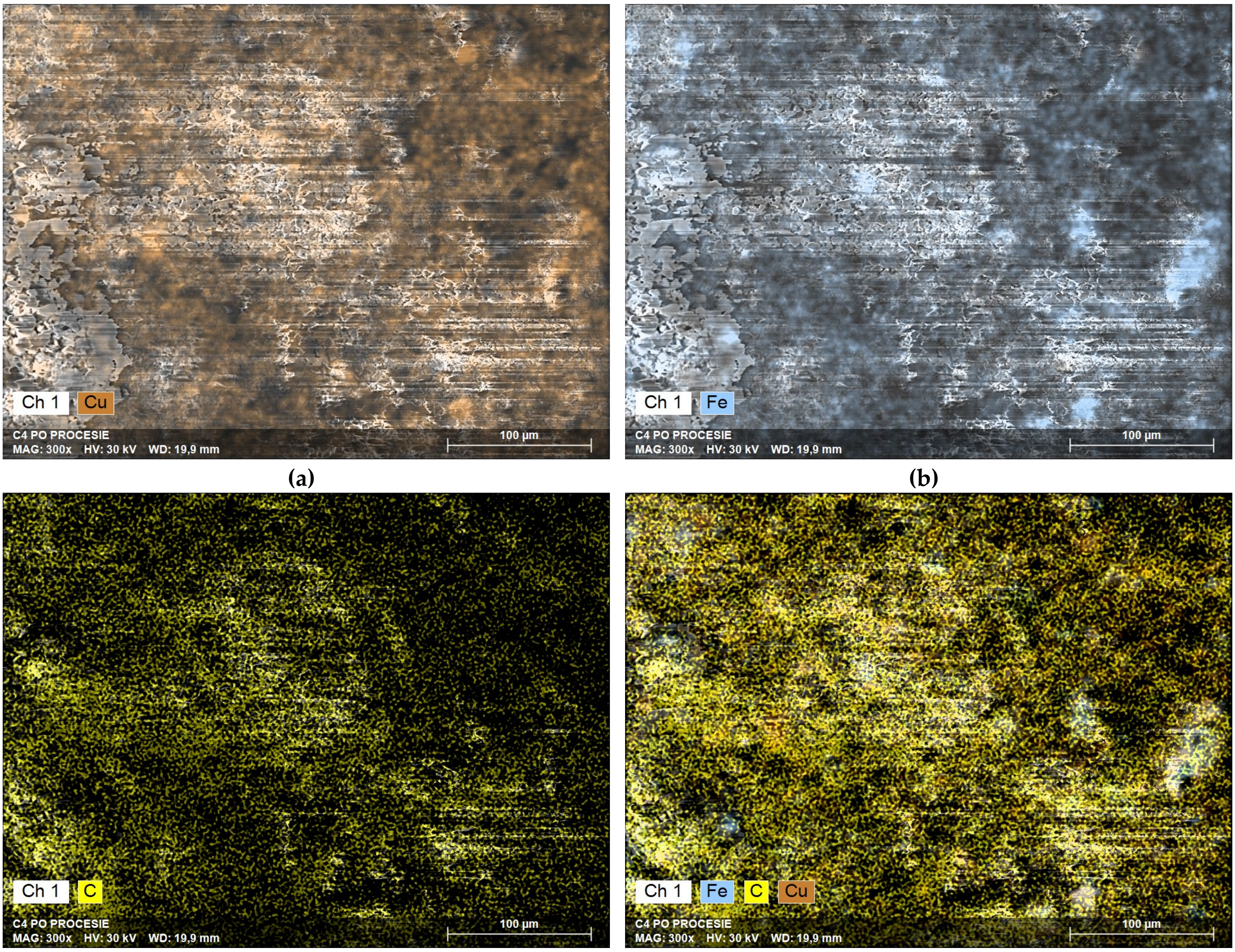

(a) **(b)** **(c)** **(d)**

Figure S13. SEM-EDS elemental mapping of the spent C4 catalyst surface showing the distribution of: (a) copper (Cu), (b) iron (Fe), (c) carbon (C), and (d) all elements superimposed (magnification 180×).

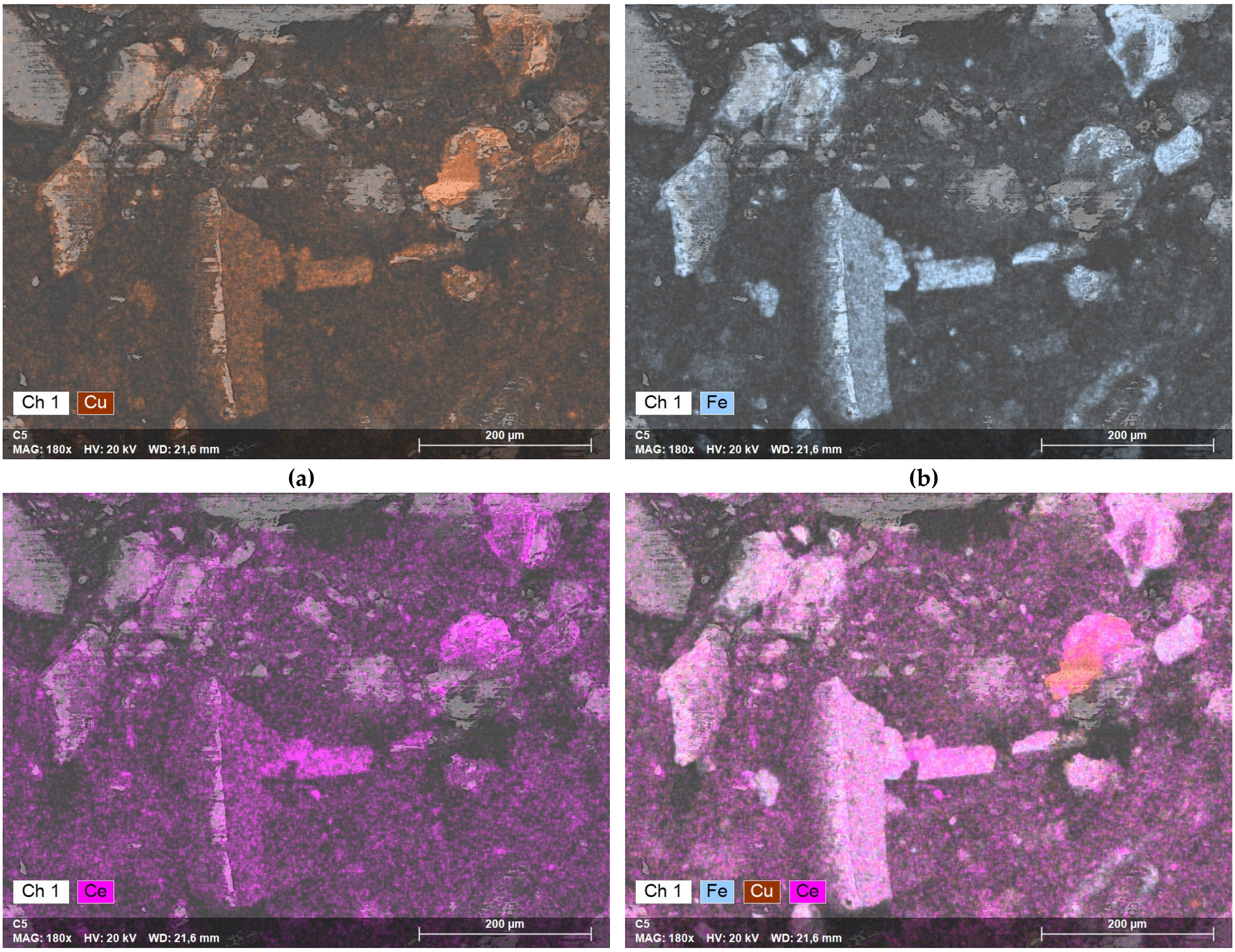

(a) **(b)** **(c)** **(d)**

Figure S14. SEM-EDS elemental mapping of the spent C5 catalyst surface showing the distribution of: (a) copper (Cu), (b) iron (Fe), (c) cerium (Ce), and (d) superimposed Fe, Cu, and Ce elements (magnification 180×).

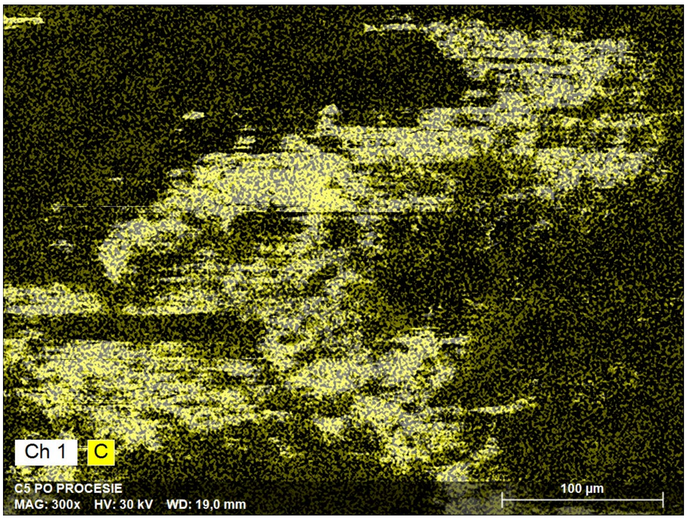

Figure S15. SEM-EDS elemental mapping showing the surface distribution of carbon (C) on the spent C5 catalyst (magnification 300×

Appendix C.2

The SEM/EDS maps were calibrated using the scale bars provided within the original images. For micrographs recorded at 300× magnification, the scale bar corresponded to 100 μm (243 px), whereas for images recorded at 180× magnification, the scale bar corresponded to 200 μm (291 px). The bottom annotation banner, legends, and scale bars were strictly excluded from the analysed area. Element-rich regions were segmented from the colour-coded EDS maps, and only connected regions larger than 50 μm² were included in the statistics to mitigate the impact of local instrumental noise. The equivalent circular diameter (ECD) was calculated based on the projected area of each detected agglomerate. The resulting statistical parameters are compiled in Table S2. To provide a comprehensive evaluation, statistics were compiled for both standalone single-element maps and multi-element overlays. The differences in the threshold-based segmentation between these two mapping regimes account for the complementary variations observed in features such as cluster counts and detected surface coverage for identical sample variants.

Table S2. Microstructural and coverage characteristics of element-rich regions determined from semi-quantitative SEM-EDS map analysis.

Variant	EDS map	Element-rich region	Map type	No. of agglomerates	Surface coverage (%)	Mean ECD ± SD (μm)	Median ECD (μm)	ECD range (μm)
B1	Figure S3a	Ni	single-element map	114	8.24	14.86 ± 8.49	11.93	8.06–57.97
B1′	Figure S4a	Ni	single-element map	17	8.22	21.51 ± 15.57	17.95	7.99–68.63
B2	Figure S5a	Ni	single-element map	44	4.51	11.69 ± 3.15	10.71	8.08–20.77
B3	Figure S6d	Ce	single-element map	47	5.15	11.93 ± 3.80	11.11	8.00–28.90
B3	Figure S7	Ni	multi-element overlay	27	3.54	12.39 ± 5.91	9.92	8.03–29.93
B3	Figure S7	Ce	multi-element overlay	24	2.98	12.25 ± 5.31	10.61	8.16–31.23
B3	Figure S6a	Ni	single-element map	42	7.94	14.55 ± 7.74	11.64	8.06–37.34
C1	Figure S9	Fe	multi-element overlay	1	0.05	8.66 ± 0	8.66	8.66–8.66
C1	Figure S9	Ni	multi-element overlay	17	2.60	12.98 ± 7.30	10.66	8.00–37.89
C1	Figure S9	Ce	multi-element overlay	16	1.26	9.94 ± 3.85	8.78	8.00–23.64
C1	Figure S8b	Fe	single-element map	42	3.11	10.04 ± 2.31	9.13	8.03–20.07
C2	Figure S10c	Cu	single-element map	36	2.83	10.26 ± 2.73	9.03	8.08–20.28
C2	Figure S10b	Fe	single-element map	16	1.33	10.66 ± 2.39	9.67	8.08–15.09
C3	Figure S12a	Cu	single-element map	47	7.86	18.49 ± 18.21	11.29	7.98–90.53

Variant	EDS map	Element-rich region	Map type	No. of agglomerates	Surface coverage (%)	Mean ECD ± SD (μm)	Median ECD (μm)	ECD range (μm)
C3	Figure S12d	Fe	multi-element overlay	14	0.40	10.25 ± 3.24	8.77	8.10–19.94
C3	Figure S12d	Cu	multi-element overlay	94	4.05	12.05 ± 5.17	10.55	7.98–43.68
C3	Figure S12b	Fe	single-element map	36	8.61	22.25 ± 21.69	14.04	8.06–101.38
C4	Figure S13a	Cu	single-element map	49	6.17	12.49 ± 4.94	10.94	7.99–30.55
C4	Figure S13b	Fe	single-element map	29	6.81	16.49 ± 8.11	15.01	8.03–48.75
C5	Figure S14c	Ce	single-element map	58	7.13	16.64 ± 14.71	12.40	8.02–88.67
C5	Figure S14a	Cu	single-element map	35	8.97	22.50 ± 23.03	12.48	7.98–115.02
C5	Figure S14b	Fe	single-element map	24	9.19	29.18 ± 26.54	20.19	8.42–128.38

Note: ECD — equivalent circular diameter. The values should be interpreted as semi-quantitative statistics of visible element-rich agglomerates rather than primary particle-size distributions. Data are extracted from both single-element maps and multi-element overlays; variations in statistical parameters for the same element within a single variant (e.g., Fe in variant C1) arise from computational segmentation thresholds inherent to single versus multi-channel signal rendering.

Appendix D

Appendix D.1

Table S3. Technical specifications, data preprocessing steps, and training hyperparameters of the developed LSTM network architecture.

Network/Training Parameter	MATLAB 2026a Code Reference	Specification and Implementation Details
Purpose of the LSTM model	Approximation of the CO_2 mole fraction at the outlet of the plasma reactor as a function of time and power number	The LSTM network was used to approximate the outlet CO_2 mole fraction as a function of time and power number.
Input data	Matrix D with dimensions n x 4	The input dataset was arranged as an n x 4 matrix.
Data structure	Column 1: time; columns 2-4: CO_2 mole fraction for PEN = 20, 3.3 and 1.65	The first column contained time, whereas the remaining columns contained the CO_2 mole fraction measured for PEN = 20, 3.3 and 1.65.
Number of sequences	Three separate training sequences	Three separate time sequences were used, corresponding to the three values of the power number.
Network input variables	Normalised time and normalised power number	The network input consisted of two features: normalised time and normalised power number.
Output variable	Normalised CO_2 mole fraction	The output variable was the normalised CO_2 mole fraction at the reactor outlet.
Data normalisation	Min-max normalisation to the range 0-1 for time, power number and CO_2 mole fraction	Time, power number and CO_2 mole fraction were normalised using min-max normalisation to the range 0-1.
Input layer	sequenceInputLayer(2)	The network contained a sequence input layer with two input features.
Number of LSTM layers	One LSTM layer	The architecture included one LSTM layer.
Number of neurons / hidden units	Parameter number_neurons, defined during the function call	The number of hidden units in the LSTM layer was specified as an adjustable training parameter.
LSTM output mode	OutputMode = 'sequence'	The LSTM layer operated in sequence-output mode, enabling prediction over the entire time sequence.
Output layer	fullyConnectedLayer(1)	A fully connected layer with one output neuron was used.
Regression layer	regressionLayer	The final layer was a regression layer for continuous-output prediction.
Complete network architecture	sequenceInputLayer(2) -> lstmLayer(number_neurons, 'OutputMode', 'sequence') -> fullyConnectedLayer(1) -> regressionLayer	The LSTM architecture consisted of a sequence input layer, one LSTM layer, one fully connected layer and a regression layer.
Activation functions	They were not defined explicitly in the code; the default mechanisms of the MATLAB lstmLayer were used	The activation functions were those implemented by default in the MATLAB LSTM layer.

Network/Training Parameter	MATLAB 2026a Code Reference	Specification and Implementation Details
Optimisation algorithm	adam	The network was trained using the Adam optimiser.
Loss function	Loss function of the regression layer; for regressionLayer, MATLAB minimises the mean squared error	The loss function was the mean squared error used by the regression layer for continuous response prediction.
Maximum number of epochs	4000	The maximum number of training epochs was set to 4000.
Mini-batch size	1	The mini-batch size was set to 1.
Initial learning rate	5×10^{-4}	The initial learning rate was 5×10^{-4}.
Gradient threshold	1	The gradient threshold was set to 1.
Data shuffling	Shuffle = 'every-epoch'	The training sequences were shuffled at every epoch.
Training set	Three sequences corresponding to PEN = 20, 3.3 and 1.65	The training set consisted of three time sequences corresponding to the investigated power numbers.
Validation set	No separate validation subset was extracted	No separate validation subset was used in this preliminary implementation.
Test set	No separate test subset was extracted	No separate test subset was used in this preliminary implementation.
Model quality assessment	RMSE calculated separately for PEN = 20, 3.3 and 1.65	The approximation quality was evaluated using RMSE calculated separately for each power number.
Comparison with ARIMA	Not performed	No comparison with an ARIMA model was carried out in this work.
Comparison with a simple RNN	Not performed	No comparison with a simple RNN architecture was carried out in this work.
Scope of interpretation	The LSTM model should be treated as a preliminary predictive tool rather than a full comparative study of time-series models	The LSTM model should be interpreted as a preliminary data-driven predictive tool, rather than as evidence of superiority over alternative time-series models.

Note. The LSTM model was used in this work as a preliminary data-driven approximation method for time-dependent DBD reactor data. Since no separate validation/test subsets or comparison with simpler models such as ARIMA or simple RNNs were included, the present results should be interpreted as a demonstration of the applicability of the LSTM architecture rather than as proof of its superiority over other predictive approaches.

https://doi.org/10.3390/xxxxx
